# Supplementary material for: Consistent Individual Differences Drive Collective Behavior and Group Functioning of Schooling Fish
Source: Curr Biol. 2017 Sep 25;27(18):2862–2868.e7. doi: 10.1016/j.cub.2017.08.004 (PMC5628957; doi:10.1016/j.cub.2017.08.004)
Supplement: Document S1. Figures S1–S4 [file mmc1.pdf]

Current Biology, Volume *27*

Supplemental Information

Consistent Individual Differences Drive Collective Behavior and Group Functioning of Schooling Fish

Jolle W. Jolles, Neeltje J. Boogert, Vivek H. Sridhar, Iain D. Couzin, and Andrea Manica

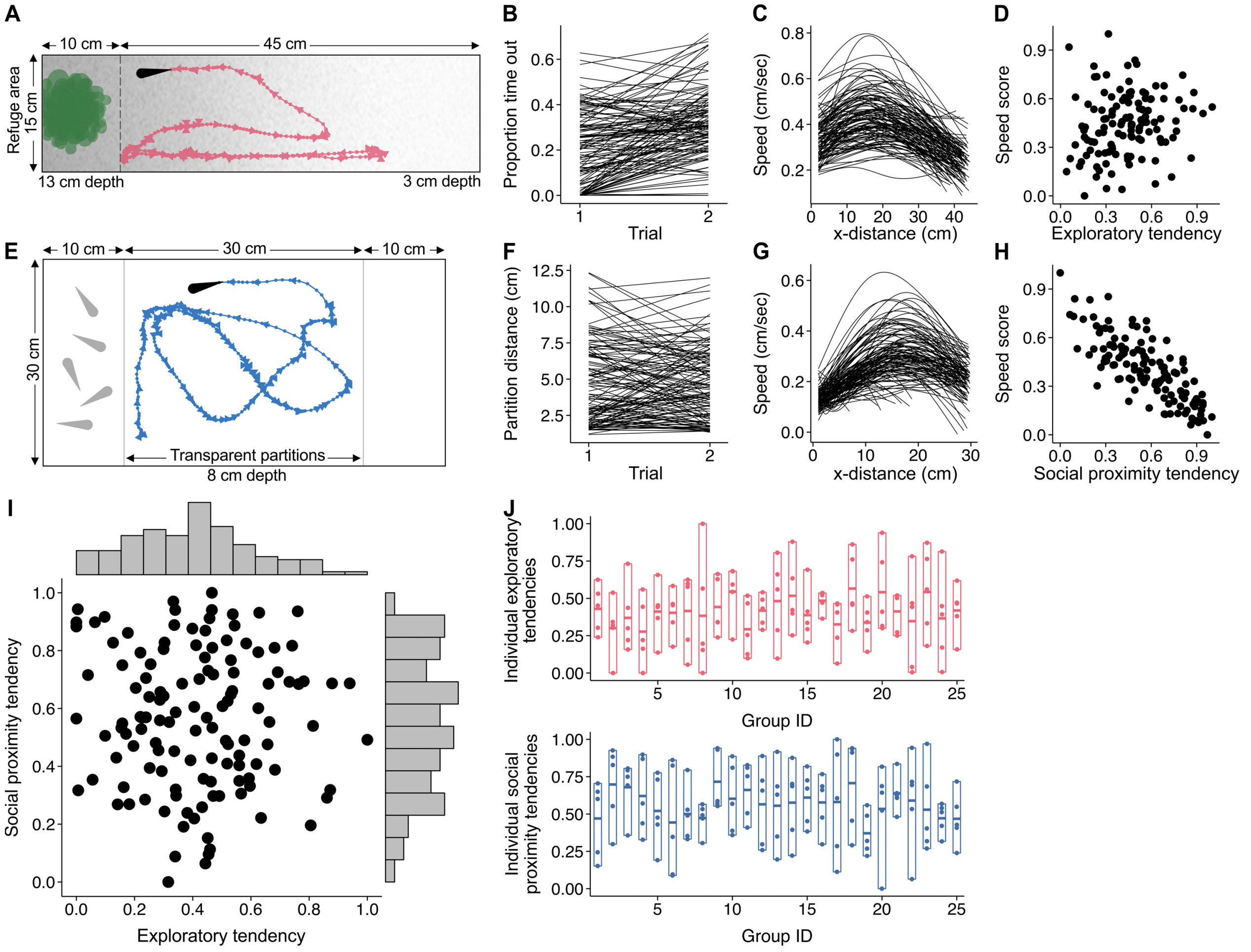

Figure S1. Consistent inter-individual behavioural differences. Related to Figure 1. (**A**) Schematic of the asocial boldness assay, a rectangular tank with a deep refuge area that leads to an increasingly shallow open area on the other side. (**B**) Line plot showing individual repeatability in terms of the proportion of time fish spent out of the refuge ('exploratory tendency'), which was strongly, positively linked to their average distance out of cover ($r_{123} = 0.67, \mathrm{p} < 0.001$). **C**) Line plot showing predicted speed curves (quadratic fit) for both boldness test trials of all fish in terms of their distance out of cover. These speed curves were used to calculate a speed score for each fish by determining the speed where the curve was maximal, averaged across both trials. **D**) Relationship between fish's exploratory tendency and their speed score (scaled) in the boldness assay ($r_{118} = 0.17, \mathrm{p} = 0.104$). (**E**) Schematic of the social assay, a tank with a large centre compartment for the focal fish, and two side compartments, one empty and one containing five conspecifics. (**F**) Line plot showing individual repeatability in terms of the average distance from the conspecifics' compartment ('social proximity tendency'). (**G**) Line plot showing predicted speed curves (quadratic fit) for both test trials of all fish in the social assay in terms of their distance from the compartment holding the shoal. (**H**) Relationship between fish's social proximity tendency and their speed score (scaled) in the social assay ($r_{123} = -0.79, \mathrm{p} < 0.001$). (**I**) Relationship between the exploratory and social proximity tendencies (n = 125 fish) and their distributions (grey bars), with behavioural scores scaled between 0 and 1. (**J**) Group compositions in terms of the individual group members' exploratory and social proximity tendencies (n = 25 groups of 5). Together, these plots show that fish were highly repeatable in their tendency to explore out of cover, as well as in their propensity to stay near the confined shoal in the sociability assay, and that no link existed between them. Fish swam faster the further they were out of cover and the further they were away from the conspecifics' compartment, towards the middle of the tank used in the two assays. While fish's exploratory tendency was only weakly linked to swim speed (**D**), fish's social proximity tendency was strongly negatively linked with swim speed (**H**), even when speed was measured in the asocial boldness assay ($r_{118} = -0.27, \mathrm{p} = 0.008$). Fish were consistent in their swim speed (speed scores) between the trials of the asocial boldness assay ($R_C = 0.41, 0.24 - 0.56$), between the trials of the social assay ($R_C = 0.58, 0.56 - 0.68$), and between the two assays ($R_C = 0.44, 0.29 - 0.56$).

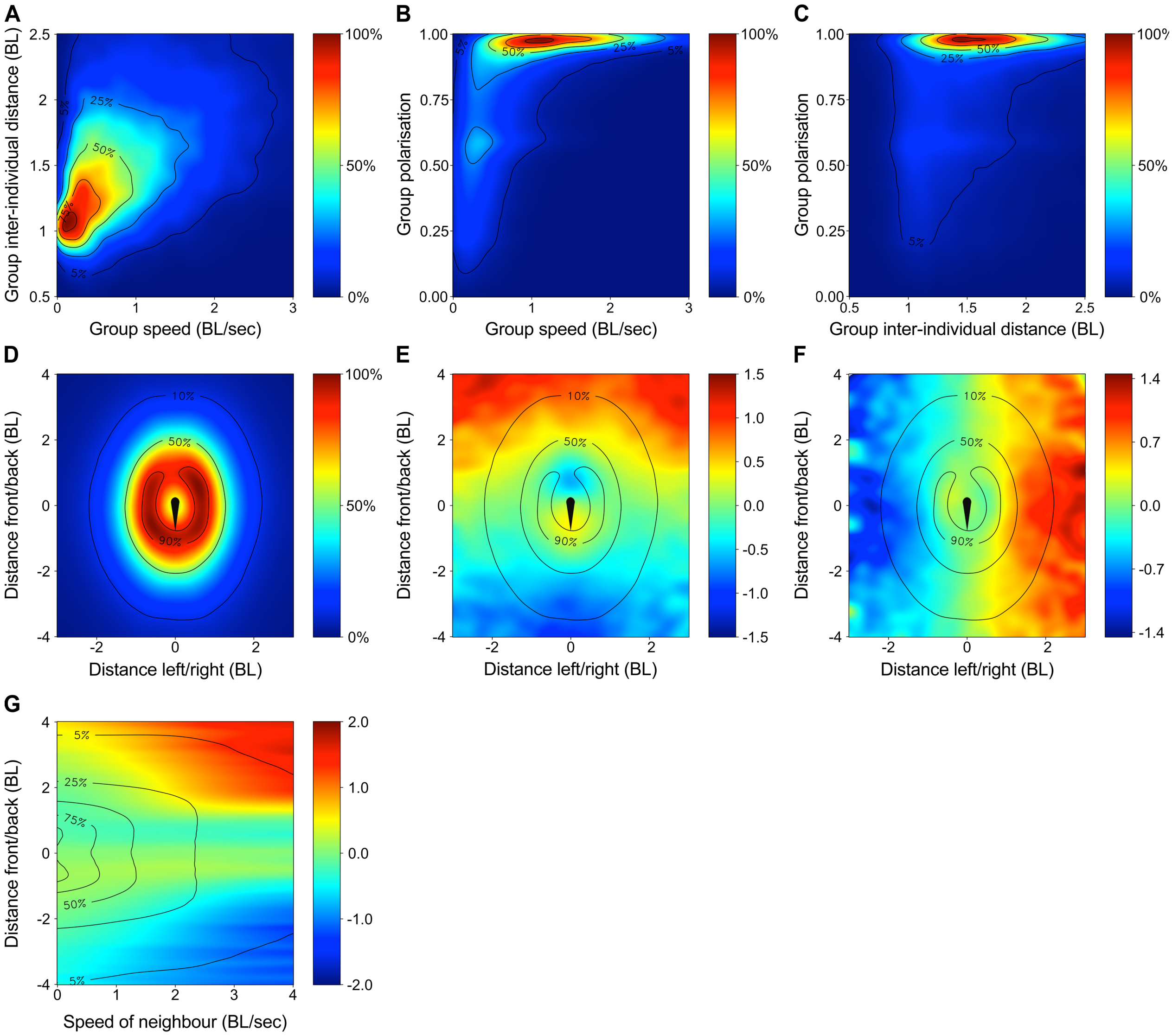

Figure S2. Heat maps of group and individual movement dynamics in the free-schooling context. Related to Figure 2 and 3. (**A**) Relationship between group speed and cohesion, (**B**) group speed and polarisation, and (**C**) group cohesion and polarisation, with group speed depicting the average median speed of the individuals in a group. Measures are expressed in units average body length (BL; 40.6 mm) where appropriate. Plots are based on the frame-by-frame data at time steps of 1/24th sec, with data cropped to show the most relevant area only (respectively 86.9%, 88.9%, and 97.5% of the full parameter space). Contours represent iso-levels in percentage of the highest bin for data of all groups combined. These plots indicate a strong link between group cohesion, speed, and polarisation, with faster moving groups being less cohesive and more strongly aligned. Groups moved at a steady median pace of 30.0 mm/sec, with an average group cohesion ($\overline{\text{IID}}$) of 70.7 mm. In the direction of motion, groups had an average length of 100 mm and rarely exceeded 300 mm. Groups were strongly polarised the majority of the time (median = 0.92) and had very low levels of fragmentation, with significant outliers or group splits (for explanation, see Methods) only occurring 4.9 ± 1.5% of the time. (**D-G**) To investigate the individual interaction rules, we selected each fish in each group and computed its position, acceleration, and turning forces relative to the position and speed of its group mates (see Methods). (**D**) The probability of finding neighbouring fish at a given position relative to the position of the focal fish, which was placed at the origin pointing north. Fish density is presented in percentages relative to the densest bin for all groups combined. (**E**) and (**F**) Respectively the acceleration and turning speed of the focal fish as a function of the position of its group mates. (**G**) Focal fish's acceleration forces as a function of the swim speed of its group mates and its front-back distance. For the turning speed, positive values indicate a right turn and negative values a left turn. Data was based on the full 30 min trial but cropped to show the most relevant area only (**D-F**: 92.1% and **G** 93.3% of the full parameter space). These plots indicate that on average, (**D**) fish are very likely to be within one body length of another group member side-by-side, and within two body lengths front-to-back ($\overline{\text{NND}}$ = 39.0 mm), (**E**) fish speed up when a neighbouring fish is far ahead or just behind them, but slow down when a neighbouring fish is far behind or just in front, (**F**) fish turn left when a neighbouring fish is on its far left side and turn right when its neighbour is on its far right side, with weaker opposite turning tendencies when neighbouring fish are very close, and (**G**) fish acceleration forces become stronger the faster the neighbouring fish is moving.

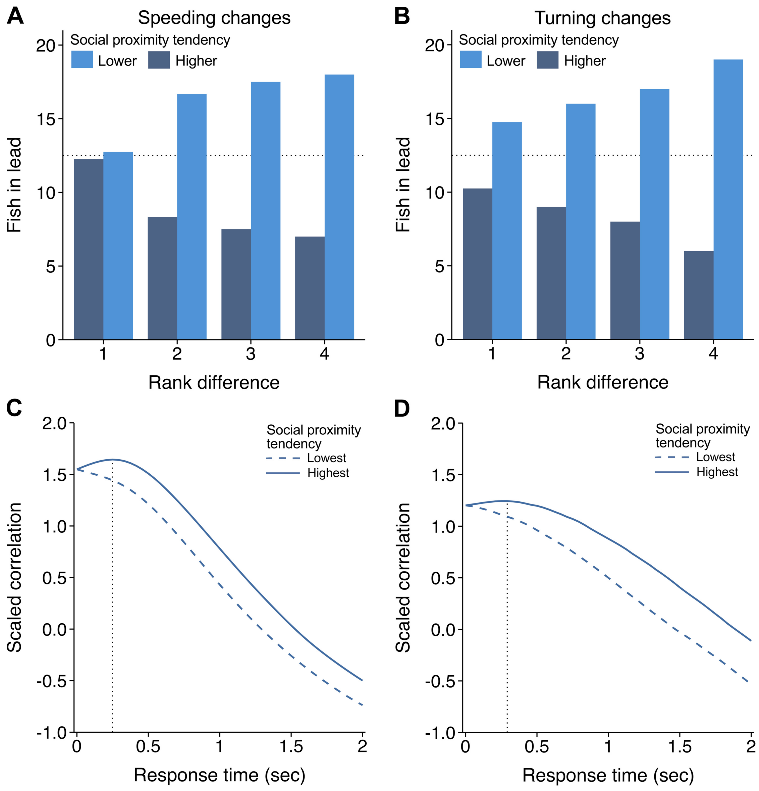

Figure S3. Propagation of movement changes in the free-schooling context. Related to Figure 2. To investigate the propagation of movement changes, we selected each fish in each group as focal individual and compared its swim speed and direction to that of all its group mates up to three seconds later, at time steps of 1/24th sec. We then determined the average time difference for the highest correlation across the trial for all dyads in each group. Fish were ranked based on their social proximity tendency (rank 1-5) within each group. (**A**) and (**B**) Bar plots depicting number of dyads for which movement changes on average propagated from the fish with the higher social proximity tendency versus from the fish with the lower social proximity tendency. Bars show mean values for rank difference of 1-3 (n = 100, 75, 50 respectively) and total number for a rank difference of 4. Dotted line represents the value that both personality ranks would lead equally. (**C**) and (**D**) Median correlations in movement changes for the fish with the highest social proximity tendency relative to fish with the lowest social proximity tendency in each group and the other way around. Correlation coefficients were scaled for each group to control for between-group variability, and analysis was restricted to frames in which both fish were moving at a speed of at least 10 mm/sec during the full 30 min trial in the free-schooling context. Both the (**C**) swim speed correlation and the (**D**) turning correlation of fish with the highest social proximity tendency in a group peaked after zero with a delay time of less than 0.5 sec before decaying (indicated by the grey dotted line), whereas for fish with the lowest social proximity tendency the correlation curve does not show such a peak. This suggests that fish with a higher social proximity tendency on average speed up, slow down and turn in response to the speed and direction of fish that have a relatively lower social proximity tendency. Both speeding, $r_{123} = 0.65, \mathrm{p} < 0.001$, and turning changes, $r_{123} = 0.54, \mathrm{p} < 0.001$, were positively linked with the tendency to be in front. These plots thus show that fish with a higher relative tendency for social proximity, which moved faster in the solitary and group assays and were more in front, are more likely to lead their group mates in terms of both (**A**) the propagation of speeding changes (ordered logistic regression: $z = -2.78, \mathrm{p} = 0.012$) and (**B**) the propagation of turning changes ($z = -2.76, \mathrm{p} = 0.012$), and that this increases the larger the rank difference between the two fish.

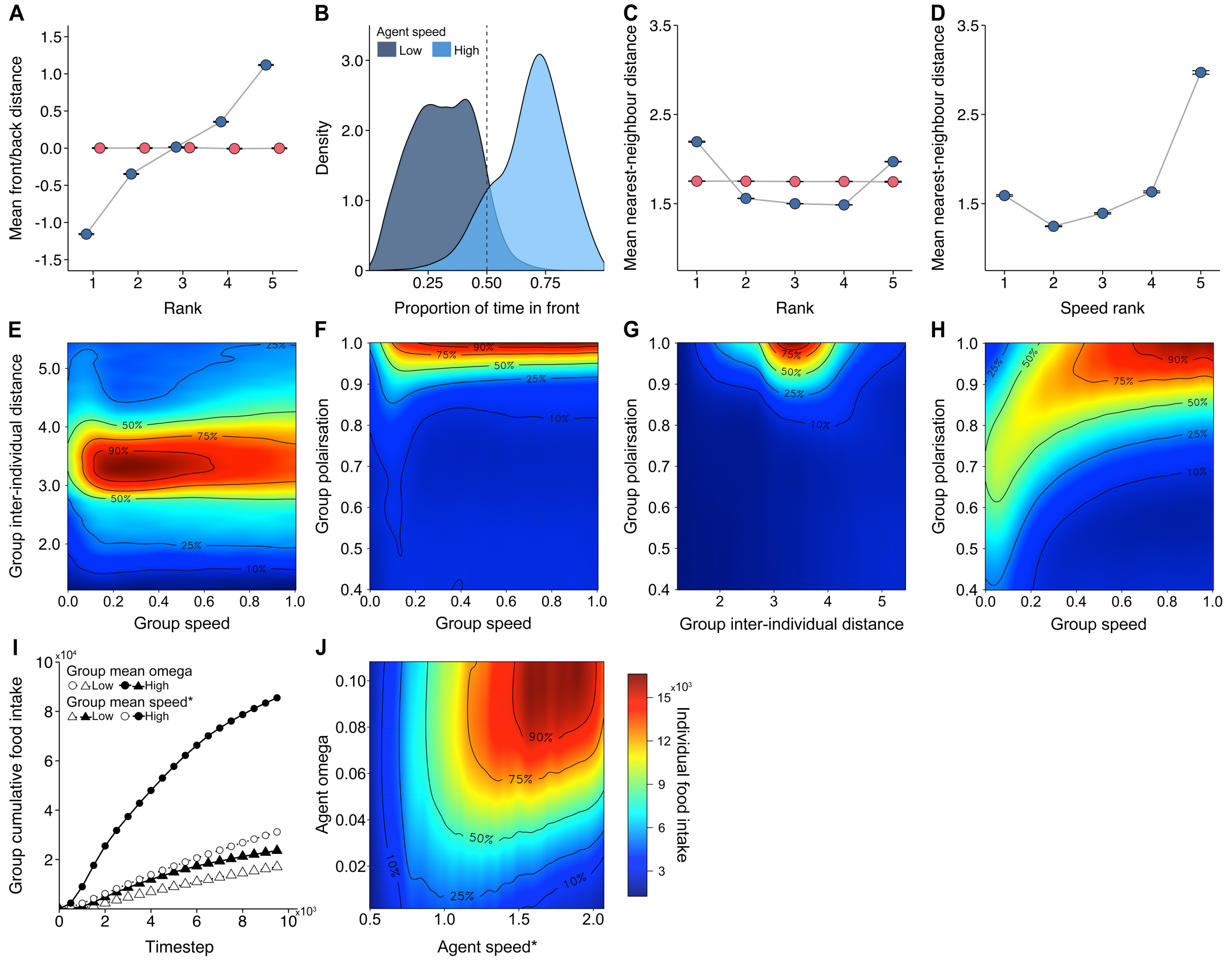

Figure S4. Data from the individual-based model simulations. Related to Figures 2, 3 and 4. (**A**) Mean distance in front/behind the group centroid in terms of an individual's speed rank (blue) or omega rank (goal-directedness; red) in the group (lower numbers indicate higher speed/omega). (**B**) Density plot of the proportion of time individuals spent in front of the group centroid in terms of their set speed, categorized in three equally sized bins with the intermediate bin not shown for clarity. (**C**) Mean nearest-neighbour distance in terms of an individual's speed rank (blue) and omega rank (red). (**D**) Mean nearest-neighbour distance in terms of an individual's speed rank but now with speed scores drawn from a Gamma distribution, see Methods and Figure S1. Front/back and nearest-neighbour distances were averaged across the simulation and expressed in units of repulsion radius. Error bars indicate 95% confidence intervals of the mean. These plots indicate that within a group, faster individuals tend to be towards the front of the group and further from their neighbours (note, also very slow individuals tend to be further away), especially when the distribution of individual speeds is right-tailed. Individual goal-directedness (omega) had no effect on these behaviours. Compare with Figure 2 and see Figure S1. (**E-H**) Surface plots depicting the relationship between (**E**) group speed and cohesion, (**F**) group speed and polarisation, (**G**) group cohesion and polarisation for groups of five individuals, and (**H**) group cohesion and polarisation for groups of 20 individuals. Plots are based on the full dataset but cropped to show the most relevant area only (respectively 90.3%, 90.4%, 92.2% and 85.8% of the full parameter space). Colour scale is square-root transformed and reflects z-scores in percentage relative to the highest bin, with contours representing iso-levels. Plots (**A-H**) are based on 400 replicates of 2,000 time steps taken at intervals of 200 time steps. Plots (**E-H**) indicate that faster groups, i.e. those composed of individuals with higher set speed, were sparser and more polarised than their slower counterparts. The link between speed and polarisation becomes especially clear when the group is larger, with groups of 20 needing higher speed to reach the same level of polarisation. The effect of speed on inter-individual distance, however, is weak. This is partly due to the three zone model, which allows for stable existence of neighbours in the alignment/orientation zone alone (see Methods), and potentially not having variable speed. Compare with Figure 3 and Figure S2. (**I**) Cumulative food intake over time, showing mean values for groups evenly split into four categories based on the average of the set distribution of speed and goal-directedness (omega). (**J**) Surface plot showing individual food intake calculated as the number of food particles consumed by an individual in terms of its speed and goal-directedness (omega). Data was cropped to show the most relevant area (73.6% of the full dataset). For comparison with fish' social proximity tendency (in Figure 4E), symbols of group speed (**I**) are inverted. Plots (**I**,**J**) are based on 400 replicates of 10,000 time steps taken at intervals of 500 time steps and indicate an interaction between individual's movemens speed and goal-orientedness drove both group and individual foraging performance: groups depleted the food more quickly the faster and more goal-oriented they were, and within groups, individuals that were faster and more goal oriented consumed more food. This is linked to the fact that faster individuals are more in front and therefore arrived at reward sites sooner than their group mates, while omega determined an individual's directedness towards the food once within the cue detection radius (see Methods). Compare with Figure 4.
