# Supplementary figures and images for: Consistent Individual Differences Drive Collective Behavior and Group Functioning of Schooling Fish
Source: Curr Biol. 2017 Sep 25;27(18):2862–2868.e7. doi: 10.1016/j.cub.2017.08.004 (PMC5628957; doi:10.1016/j.cub.2017.08.004)

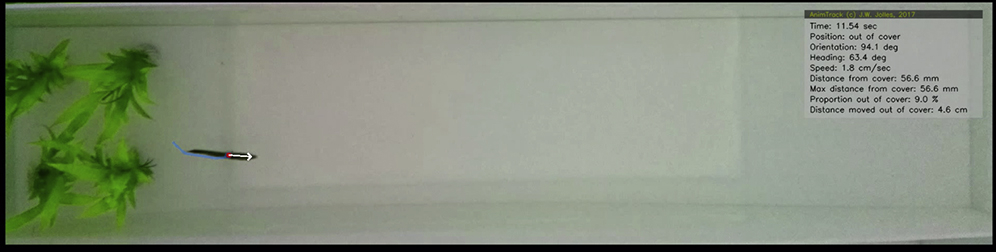

Supplement: Movie S1. Individual Personality Assays, Related to Figures 1 and S1 — Movie showing the tracking of an individual fish in the classic boldness and sociability assays with the automatically computed behavioral measures. [file mmc2.jpg]

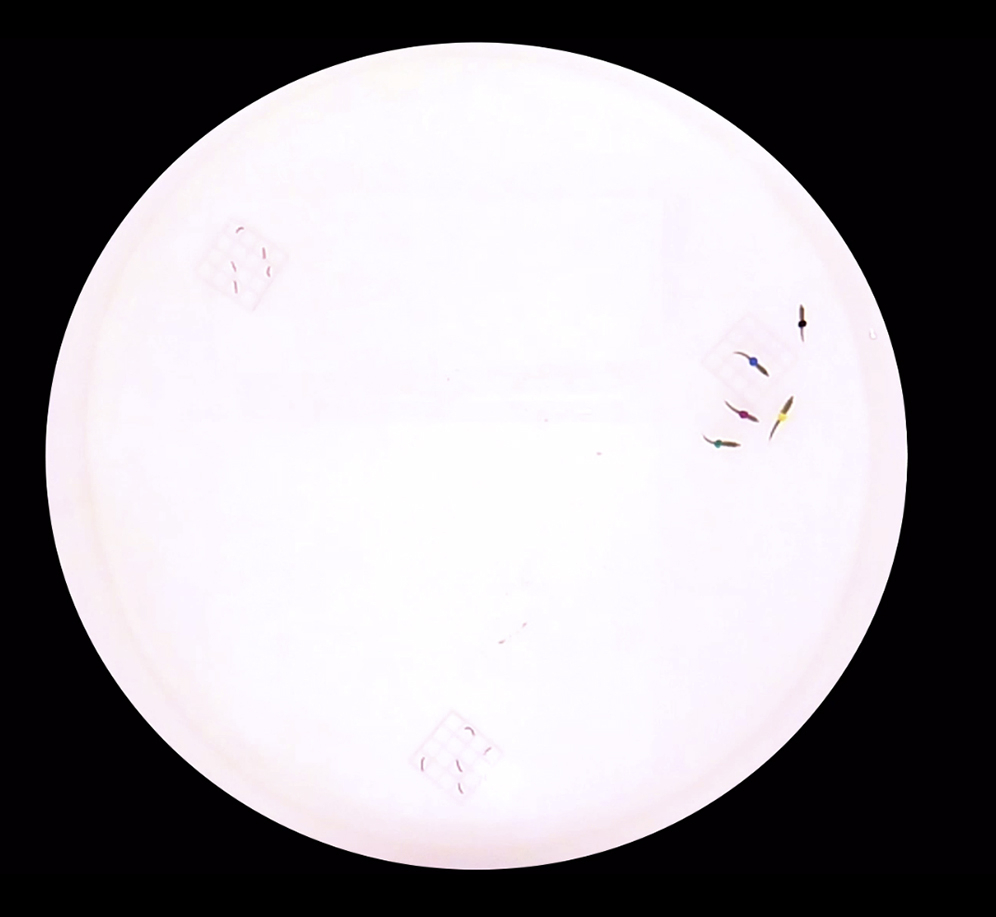

Supplement: Movie S2. Group Shoaling Experiments, Related to Figures 1 and S2 — Movie showing a group of fish in each of the three assays used for the group experiments: the free-schooling context, the open foraging context, and the semi-covered foraging context. [file mmc3.jpg]

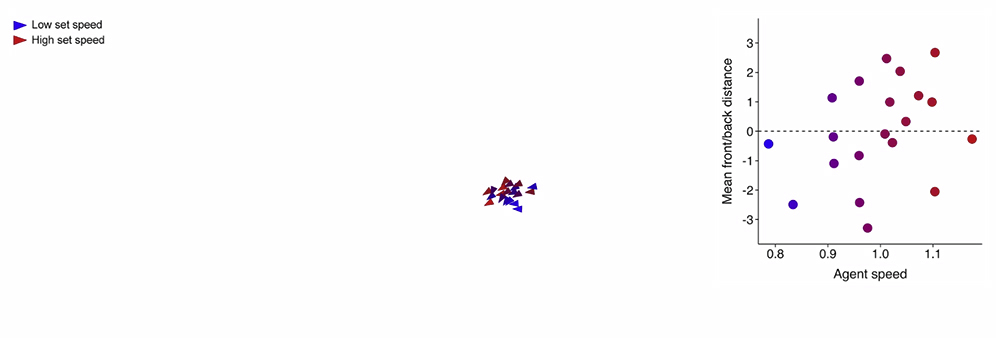

Supplement: Movie S3. Individual-Based Simulations of Self-Organizing, Heterogeneous Groups, Related to Figure S4 — Movie depicting a visualization of the individual-based simulations of self-organized groups consisting of 5 and 20 agents that differ in their set speed, with the emergence of spatial leadership plotted dynamically over time. [file mmc4.jpg]
