# Supplementary material for: Consistent Individual Differences Drive Collective Behavior and Group Functioning of Schooling Fish
Source: Curr Biol. 2017 Sep 25;27(18):2862–2868.e7. doi: 10.1016/j.cub.2017.08.004 (PMC5628957; doi:10.1016/j.cub.2017.08.004)
Supplement: Document S2. Article plus Supplemental Information [file mmc5.pdf]

Report

Current Biology

Consistent Individual Differences Drive Collective Behavior and Group Functioning of Schooling Fish

Highlights

- Fish with low sociability scores swim faster in both social and asocial contexts
- This trait explains the structure, leadership, dynamics, and coordination of groups
- Together with boldness, it in turn drives individual and group foraging success
- These patterns emerge naturally in simulations of self-organized groups

Authors

Jolle W. Jolles, Neeltje J. Boogert, Vivek H. Sridhar, Iain D. Couzin, Andrea Manica

Correspondence

j.w.jolles@gmail.com

In Brief

Jolles et al. demonstrate in schooling fish that consistent individual differences drive the structure, leadership, movement dynamics, and foraging performance of groups. These effects emerge naturally from simulations of self-organized, heterogeneous groups. This study shows that individual differences provide a mechanism to explain collective behavior.

Jolles et al., 2017, Current Biology *27*, 2862–2868
September 25, 2017 © 2017 The Author(s). Published by Elsevier Ltd.
http://dx.doi.org/10.1016/j.cub.2017.08.004

CellPress
Current Biology
Report

Consistent Individual Differences Drive Collective Behavior and Group Functioning of Schooling Fish

Jolle W. Jolles,[1,2,3,5,*] Neeltje J. Boogert,[1,4] Vivek H. Sridhar,[2,3] Iain D. Couzin,[2,3] and Andrea Manica[1]
[1]Department of Zoology, University of Cambridge, Downing Street, Cambridge CB2 3DT, UK
[2]Department of Collective Behaviour, Max Planck Institute for Ornithology, Am Obstberg 1, Radolfzell 78315, Germany
[3]Department of Biology, University of Konstanz, Universitätsstrasse 10, Konstanz 78464, Germany
[4]Centre for Ecology and Conservation, University of Exeter, Penryn Campus, Penryn, Cornwall TR10 9FE, UK
[5]Lead Contact
*Correspondence: j.w.jolles@gmail.com
http://dx.doi.org/10.1016/j.cub.2017.08.004

SUMMARY

The ubiquity of consistent inter-individual differences in behavior ("animal personalities") [1, 2] suggests that they might play a fundamental role in driving the movements and functioning of animal groups [3, 4], including their collective decision-making, foraging performance, and predator avoidance. Despite increasing evidence that highlights their importance [5–16], we still lack a unified mechanistic framework to explain and to predict how consistent inter-individual differences may drive collective behavior. Here we investigate how the structure, leadership, movement dynamics, and foraging performance of groups can emerge from inter-individual differences by high-resolution tracking of known behavioral types in free-swimming stickleback (*Gasterosteus aculeatus*) shoals. We show that individual's propensity to stay near others, measured by a classic "sociability" assay, was negatively linked to swim speed across a range of contexts, and predicted spatial positioning and leadership within groups as well as differences in structure and movement dynamics between groups. In turn, this trait, together with individual's exploratory tendency, measured by a classic "boldness" assay, explained individual and group foraging performance. These effects of consistent individual differences on group-level states emerged naturally from a generic model of self-organizing groups composed of individuals differing in speed and goal-orientedness. Our study provides experimental and theoretical evidence for a simple mechanism to explain the emergence of collective behavior from consistent individual differences, including variation in the structure, leadership, movement dynamics, and functional capabilities of groups, across social and ecological scales. In addition, we demonstrate individual performance is conditional on group composition, indicating how social selection may drive behavioral differentiation between individuals.

RESULTS AND DISCUSSION

In recent years, it has become apparent that across a wide range of animal taxa, individuals commonly differ consistently from one another in their behavior [1, 2] ("animal personalities"), often with large fitness consequences [17] and wide-ranging ecological and evolutionary implications [18, 19]. Such variation could provide a level of heterogeneity within animal groups that may drive collective behavior. Indeed, recent studies have started to provide support for that notion and have shown that consistent behavioral differences can influence leadership [5–8], social network structure [9, 10], collective dynamics [11, 12], and group performance [13–16]. However, rarely are consistent behavioral differences integrated within the mechanistic framework of collective behavior research [12, 20], which has demonstrated that relatively simple interaction rules play an important role in the emergence of collective behavior [21–23]. It therefore remains unclear how consistent individual differences in behavior drive the structure, movement dynamics, and functioning of animal groups.

Here, we combine high-resolution tracking of individuals with known behavioral types in free-swimming stickleback (*Gasterosteus aculeatus*) shoals, with agent-based models of self-organizing groups, to provide a more mechanistic and predictive understanding of the behavior, structure, and performance of groups across ecological contexts. To capture the essential dynamics within and between groups, we employ a deliberately simple, spatially explicit model, which has previously been used successfully to explain the emergence of leadership, group structure, and consensus decision-making in a range of species [12, 24–27].

We first determined the behavioral tendencies of 125 fish by exposing them to two classic personality assays and tracking their movements (see Figure S1). We found consistent inter-individual variation in fish's tendency to leave a refuge and explore an open environment (repeatability R_C = 0.48, 95% confidence intervals: 0.33–0.60). This exploratory tendency, which is traditionally referred to as "boldness" since it may increase potential predation risk [28], was positively linked to individuals' food consumption even in the safety of the holding compartment [29], reflecting an intrinsic higher motivation for food. We also found consistent individual differences in fish's proximity to a confined shoal of conspecifics (R_C = 0.58, 0.46–0.68), classically used to define "sociability" [30, 31],

 © 2017 The Author(s). Published by Elsevier Ltd.
This is an open access article under the CC BY license (http://creativecommons.org/licenses/by/4.0/).

CellPress

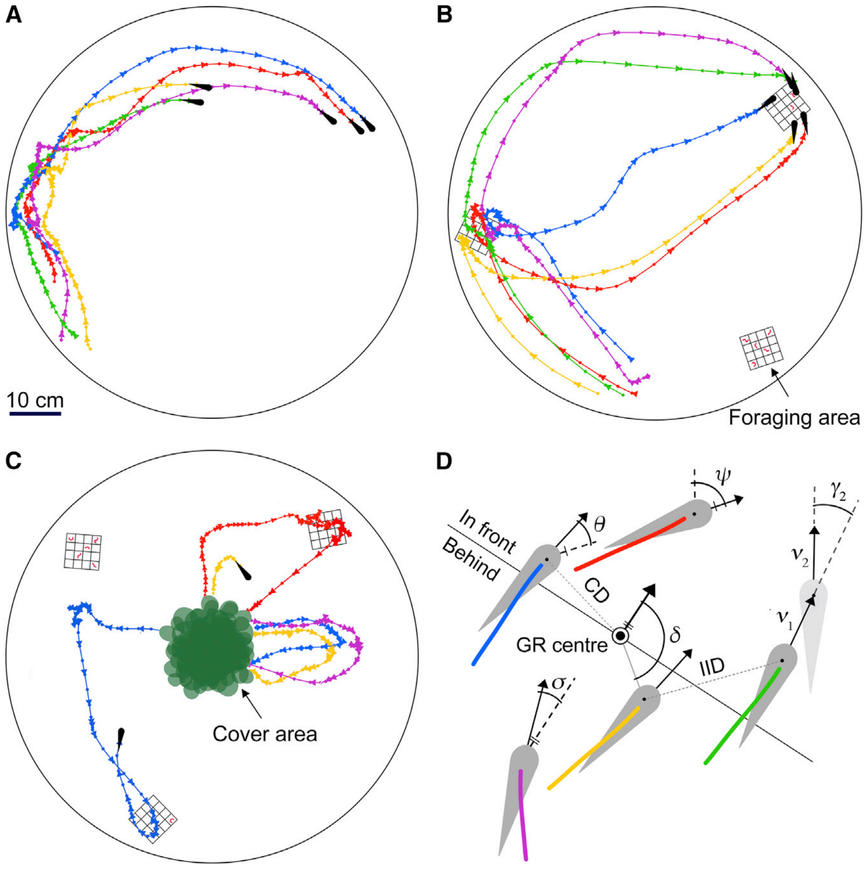

Figure 1. Group Shoaling Experiments

(A–C) Schematics of (A) the free-schooling context, (B) the open foraging context with patches of food, and (C) the semi-covered foraging context with patches of food and plant cover. Schematics show tracking segments of one randomly selected group, with colors corresponding to the individual fish. Triangles point in the direction of motion.

(D) Graphic illustrating key spatial and movement characteristics with arrows depicting movement vectors. For the individual assays, see Figure S1.

which was not correlated with their exploratory tendency (r_{123} = −0.05, p = 0.658). Based on the detailed tracking data, we found that individual fish slowed down the closer they were to the confined shoal and that fish that consistently stayed closer to the shoal also swam at consistently lower speeds. This was even the case when controlling for boundary effects (r_{123} = −0.79, p = 0.001) and when measured in the asocial boldness assay (see Figure S1). These results show that a fundamental link exists between social proximity and speed and concord with the general observation that slow-moving individuals tend to form more cohesive groups [25]. As consistent differences in social proximity can thus potentially be both a cause and a result of differences in speed, we prefer to refer to this trait as fish's "social proximity tendency."

After quantifying the behavioral tendencies of the fish, we tagged all individuals for identification (see STAR Methods) and allocated them randomly to groups of five (n = 25 groups; see Figure S1). In their natural habitat, animals may experience open, homogeneous spaces, encounter resources in spatial and temporal patches, and use habitat structures to hide from predators [30, 31]. We therefore tested the groups repeatedly in three contexts that reflect these different, ecologically relevant scenarios, each set up in the same large, circular tanks (Figures 1A–1C). Using custom-written software, we automatically identified and tracked the position of each fish in the freely moving groups and computed fine-scale spatial, movement, and foraging data (Figure 1D; see STAR Methods).

On average, sticklebacks moved in highly cohesive, ordered shoals and maintained clear zones of attraction and repulsion, mediated by relative changes in their speed and heading (Figure S2), in high accordance with other fish species [32, 33]. However, large and consistent differences existed between the 25 groups in terms of their structure and movement dynamics. To investigate how this variability could be explained by the behavioral tendencies of individuals within the groups, we employed a linear mixed modeling approach (see STAR Methods).

We first exposed the groups to the conventional collective scenario [23], free movement within an open, homogeneous environment (Figure 1A). The speed that fish adopted in the freely moving groups was positively linked to their speed in the individual personality assays (χ^2 = 7.84, p = 0.012), and individuals with lower social proximity tendencies, which had higher speeds in the individual assays, swam significantly faster in this group context (χ^2 = 8.70, p = 0.009). Fish also strongly conformed in their speed (c.f. [34]), a requirement to maintain group cohesion, and, on average, slowed down or sped up when grouped with others that had, respectively, a high or low mean social proximity tendency (χ^2 = 7.68, p = 0.012).

In terms of spatial positioning, fish had smaller nearest-neighbor distances the higher their social proximity tendency (χ^2 = 26.79, p < 0.001; Figure 2A). As a result of relative differences within groups, it was the fish with relatively low social proximity tendencies (which were also faster) who occupied positions toward the periphery (χ^2 = 29.98, p < 0.001; Figure 2B) and front (Figure 2C) of their group, an effect that strengthened over time (5 min: ΔAIC = 38.59 versus 30 min: χ^2 = 9.14, p = 0.008). This result is in line with theory [24] and recent work on pigeons [8] that show that faster individuals tend to lead. By assessing the propagation of movement changes in the groups [35], we further found that such faster-moving, leading fish with lower social proximity tendencies were also much more influential in deciding group motion (Figure S3) and that, as a result, directional leader-follower networks emerged (Figure 2D). These findings suggest a potential self-organizing mechanism for the emergence of group structure and leadership from individual

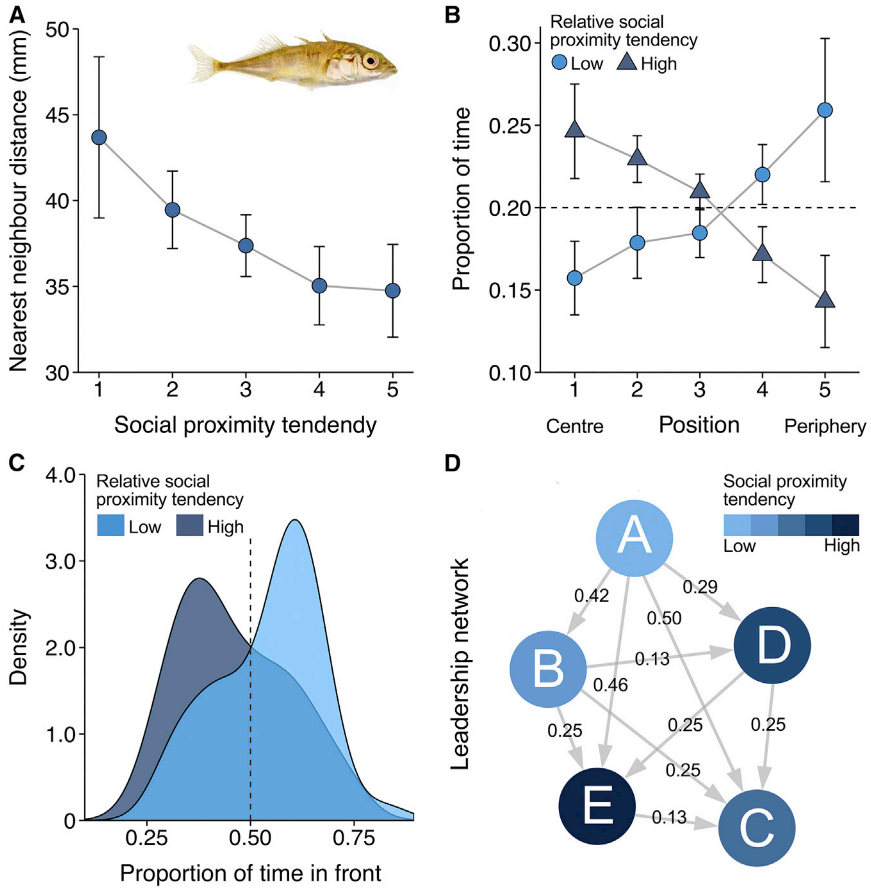

Figure 2. Effect of Social Proximity Tendency on Spatial Positioning and Leadership

(A) Fish nearest neighbor distance in groups as a function of their social proximity tendency, shown in five equally sized categories (mean ± 2 SEM; n = 120 fish).

(B) Proportion of time fish occupied the most central to the most peripheral position in the group, calculated for each frame and averaged per individual across all frames (mean ± 2 SEM).

(C) Density plot of the proportion of time individuals spent in front of the group center for the full 30 min trial.

(D) Visualization of a leadership network in terms of propagation of speeding changes of one randomly selected group. Numbers indicate the average temporal delay in seconds and arrows point in the direction of propagation; see Figure S3.

For plots (B) and (C), individuals were evenly distributed into three categories, with the intermediate category not shown for clarity. All data were analyzed as a continuous variable. See also Figures S2 and S4 for model simulations.

differences in speed, with individuals' behavior being determined by their own tendencies as well as the tendencies of other group members. In the open, homogeneous environment, fish's exploratory tendency had no effect on either spatial positioning (center distance rank: $\chi^2 = 0.64$, $p = 0.495$) or leadership (proportion of time in front: $\chi^2 = 0.06$, $p = 0.804$).

From the behavioral tendencies of the individual fish, large differences in structure and movement dynamics also emerged between the groups. When together as a group, those shoals of individuals with, on average, low social proximity tendencies (and thus high individual speeds) moved relatively quickly, with high alignment and spacing between individuals, and predominantly schooled (Figure 3; $r_s = -0.52$, $p = 0.014$). In contrast, shoals with a high mean social proximity tendency moved relatively slowly and with little alignment but were much more cohesive ($F_{1,22} = 9.31$, $p = 0.012$; Figure 3). Further, when measuring the strength of social interactions in the groups, we found the strongest social forces (i.e., stronger responsiveness) were exhibited in the fastest-moving groups (Figure S2G; c.f. [32]). This suggest that groups that would conventionally be labeled as highly sociable based on the classic assay actually have the weakest social forces, linked to their low speeds, highlighting the need for a mechanistic assessment and careful terminology when considering individual and group behavior. As for individual spatial positioning and leadership, the exploratory tendencies of the fish also had no effect on the cohesion ($F_{1,22} = 1.51$, $p = 0.305$) or schooling dynamics ($r_s = 0.23$, $p = 0.337$) of the groups.

To relate our experimental results to theory and to seek a parsimonious explanation for the observed patterns, we conducted simulations of a generic model of self-organized groups. We integrated consistent individual differences in the classic parameters of speed and goal-orientedness (ω), defined as the likelihood that an individual biases its motion toward a desired goal rather than respond to social information [24, 27]. We found that this simple agent-based model qualitatively recreated the patterns observed experimentally, both in terms of fish's social proximity tendency driving the spatial positioning and leadership of individuals and the structure and movement dynamics of groups and in terms of the lack of such effects for fish's exploratory tendency (Figure S4).

Building on previous work [8, 25, 32], our study combines empirical data from individual and group assays with model simulations to provide evidence that heterogeneity in speed is a causal mechanism that drives group states, including the structure, leadership, cohesion, and alignment of groups. Due to differences in swim speed, faster group members passively arrive at positions near the edge and front of groups, which in turn increases their propensity to lead. At the same time, higher individual speeds increase the speed of the group, which thereby passively results in higher order (alignment) and spacing between individuals. Differences in individual speed can be intrinsic or an emergent property, both of other intrinsic (e.g., size) and labile (e.g., nutritional state) characteristics, as well as external factors (e.g., predation risk). These results thus provide a relatively simple candidate mechanism by which collective behavior can emerge passively from individual differences without the need for global knowledge. Our finding that social proximity was strongly, negatively linked with speed across social and asocial contexts warrants further work to investigate

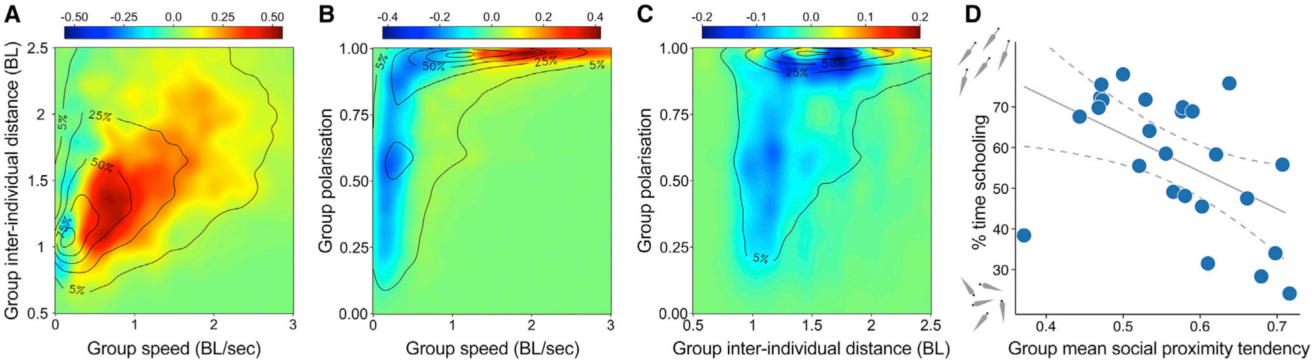

Figure 3. Group Structure and Movement Dynamics in Relation to Group Mean Social Proximity Tendency

(A–C) Heatmaps showing the distribution and link between the three key components of collective motion for groups with a low mean social proximity tendency (n = 13) relative to groups with a high mean social proximity tendency (n = 12). Groups with a relatively high social proximity tendency were more likely to be found in the bluer regions of the plots, whereas groups with relatively low social proximity tendency were more likely to be found in the redder regions of the plots. Group speed depicts the mean median swimming speed of the individuals in a group and is qualitatively similar to the speed of the group centroid. Plots are based on frame-by-frame data at time steps of 1/24th s, with groups evenly allocated to two categories based on their mean social proximity tendency. Units are in mean body length (BL; 40.6 mm), and contours represent iso-levels in percentage of the highest bin for all groups combined; see Figure S2.

(D) Proportion of time groups were schooling, characterized based on the raw distributions of group speed, cohesion, and polarization (see STAR Methods). Solid gray line and dashed gray lines indicate a linear fit to the data with 95% confidence intervals.

the extent that consistency in social proximity, classically termed "sociability," is driven by an intrinsic social tendency rather than the preferred movement speed of individuals.

To further investigate the functional consequences of the behavioral tendencies of individuals within groups, we exposed the shoals to an open and to a semi-covered environment with patches of food (Figures 1B and 1C; see STAR Methods) and analyzed group foraging dynamics and performance. Fish with a low social proximity tendency (which tended to move relatively fast) were most likely to first discover the foraging areas in the open foraging context (Figure 4A), in line with their tendency to be in front (see Figure 2C), whereas in the semi-covered foraging environment it was highly exploratory fish that made most discoveries (traits × context: χ^2 = 5.77, p = 0.030). After the discovery of the food, it was exploratory fish that were fastest to actually consume the food, both in the open and in the semi-covered foraging environment (survival model [SM]: z = 3.63, p = 0.001; Figure 4B). Due to the availability of cover, individuals spent considerable time hiding and groups often split, with exploratory fish being the most likely to initiate foraging trips and thereby lead their group-mates out of cover (χ^2 = 8.15, p = 0.011), but also to spent more time out of cover alone (χ^2 = 10.28, p = 0.005; Figure 4C), a behavior that may lead to higher potential predation risk [28, 30].

Ultimately, it was the combined effects of fish's social proximity and exploratory tendencies that explained the foraging performance of both groups and individuals. Overall, groups composed of exploratory fish that had a low social proximity tendency (and thus moved relatively fast) found and depleted the food patches most quickly (SM: z = −2.20, p = 0.046), with the relative effect of fish's exploratory tendency intensified by the availability of cover (z = 3.15, p = 0.006; Figure 4E). The interaction of both traits also predicted the foraging performance of the individual fish, with again the relative tendencies (rather than the absolute tendencies) being important (ΔAIC = +13.94): exploratory fish with low social proximity tendencies had the highest food intake, with the food intake of more exploratory fish being enhanced in the semi-covered environment (traits × context: χ^2 = 10.32, p = 0.005; Figure 4F). Overall, fish with low social proximity tendencies experienced greater variance in food intake ($F_{41,39}$ = 2.06, p = 0.044; Figures 4D and 4F), in line with the prediction that leadership positions come with higher variance in fitness [36].

Again, the general effects of the behavioral tendencies of the fish, here on the foraging performance of both individuals and groups, emerged naturally in simulations of our agent-based model: groups with high mean speed and goal-orientedness depleted food patches most quickly, and individuals with a high speed and a goal-oriented tendency had the highest food intake (Figure S4). These findings show that the exploratory or "boldness" tendency of individuals is intrinsically linked to their goal-directedness and motivation for food [5, 16, 29] and thereby drives foraging performance directly, whereas the social proximity tendency of individuals had an indirect effect on foraging performance by the effects of speed.

In summary, we present results from detailed behavioral experiments on individuals and groups of fish in combination with agent-based model simulations that demonstrate how collective behavior can emerge from consistent inter-individual differences, including spatial positioning and leadership within groups, differences in structure and movement dynamics between groups, and group and individual foraging performance. Individual differences in speed and goal-orientedness provide a simple, self-organizing mechanism by which collective behavior and group functioning can emerge without individuals requiring global knowledge of their group. These findings provide fundamental insights that may help explain and ultimately predict the emergence of complex collective behavioral patterns across social and ecological scales. We also show that the spatial positioning, leadership, and foraging performance of individuals was conditional on the composition of their

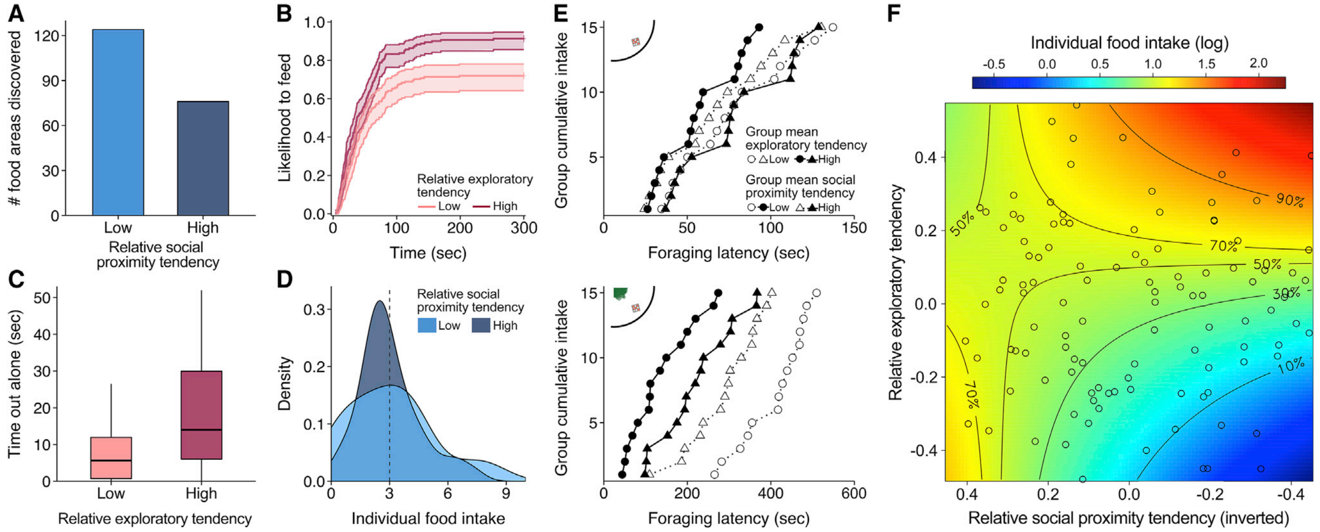

Figure 4. Effects of Individual Social Proximity and Exploratory Tendencies on Group Foraging Dynamics

(A) Total number of foraging areas discovered during the open foraging context trials (out of 295 discoveries).

(B) Inverted survival plot with confidence intervals of fish's likelihood to feed in the open and semi-covered foraging context.

(C) Boxplots depicting total time spent out of plant cover alone in the semi-covered foraging context when food was still available.

(D) Density plot of the mean number of food items eaten per trial across both foraging contexts.

For plots (A)–(D), individual tendencies were evenly distributed into low, medium, and high categories (n = 42, n = 42, and n = 41 fish, respectively), with the intermediate category not shown for clarity.

(E) Group foraging speed in the open (top) and semi-covered foraging cover context (bottom) in terms of the latency to consume each food item (15 provided per trial). The plot shows latencies averaged across trials for each group, and groups split into four categories based on their mean exploration and social proximity tendencies (low-low, low-high, high-low, and high-high: n = 5, 8, 8, and 4, respectively).

(F) Surface plot of the mean number of food items eaten (log transformed) in the open foraging context (points indicate individual fish), based on a generalized linear mixed model (GLMM) fit to the data, cropped to 90% to show the effect excluding fish with the most extreme tendencies (n = 12 fish). Relative social proximity tendency is shown inverted such that faster fish are on the right and slower fish on the left, directly comparable with the model simulations of speed (see Figure S4).

group. Over time, this could result in behavioral feedback loops that may lead to behavioral differentiation between individuals via social selection [37], which may help explain the evolutionary maintenance of personality types [36, 37]. Our study calls for a new generation of theoretical and empirical work that further integrates individual differences with collective behavior to better understand the multi-scale consequences of consistent behavioral variation, from within-group positioning to group formation and population dynamics [37, 38], as well as its potential drivers, via group-dependent effects on individual performance.

STAR★METHODS

Detailed methods are provided in the online version of this paper and include the following:

- KEY RESOURCES TABLE
- CONTACT FOR REAGENT AND RESOURCE SHARING
- EXPERIMENTAL MODEL AND SUBJECT DETAILS
- METHOD DETAILS
  - Experimental overview
  - Individual behavioral assays
  - Group shoaling experiments
  - Automated tracking and data collection
  - Individual-based modeling
- QUANTIFICATION AND STATISTICAL ANALYSIS
  - Computation of behavioral data
  - Data analysis
- DATA AND SOFTWARE AVAILABILITY

SUPPLEMENTAL INFORMATION

Supplemental Information includes four figures and three movies and can be found with this article online at http://dx.doi.org/10.1016/j.cub.2017.08.004.

AUTHOR CONTRIBUTIONS

The study was conceived by J.W.J. and A.M with important input from N.J.B. J.W.J. and N.J.B performed the experiments, J.W.J analyzed the data, V.H.S. and I.D.C. performed the model simulations and provided extensive additional input. J.W.J drafted the manuscript with substantial contributions from all other authors.

ACKNOWLEDGMENTS

We thank Lucy Aplin, Damien Farine, Matt Grobis, Rufus Johnstone, Jelle Jolles, Kate Laskowski, Máté Nagy, Mike Webster, and Peter Winsberger for discussions or comments on the manuscript, and Ben Walbanke-Taylor for logistical support. We would also like to thank two anonymous reviewers for their constructive feedback. We acknowledge financial support from the Biotechnology and Biological Sciences Research Council (graduate studentship to J.W.J), the Association for the Study of Animal Behaviour (research grants to J.W.J and N.J.B), the Royal Society (Dorothy Hodgkin Fellowship to N.J.B), the National Science Foundation (IOS-1355061 and

EAGER-IOS-1251585 to I.D.C), the Office of Naval Research (N00014-09-1-1074 and N00014-14-1-0635 to I.D.C), the Army Research Office (W911NG-11-1-0385 and W911NF-14-1-0431 to I.D.C), the Human Frontier Science Program (RGP0065/2012 to I.D.C), the Struktur- und Innovationsfonds für die Forschung of the state Baden-Württenberg (SI-BW; to I.D.C), and the Max Planck Society.

Received: April 24, 2017
Revised: June 20, 2017
Accepted: August 2, 2017
Published: September 7, 2017

REFERENCES

1. Réale, D., Reader, S.M., Sol, D., McDougall, P.T., and Dingemanse, N.J. (2007). Integrating animal temperament within ecology and evolution. Biol. Rev. Camb. Philos. Soc. *82*, 291–318.
2. Bell, A.M., Hankison, S.J., and Laskowski, K.L. (2009). The repeatability of behaviour: a meta-analysis. Anim. Behav. *77*, 771–783.
3. Webster, M.M., and Ward, A.J.W. (2011). Personality and social context. Biol. Rev. Camb. Philos. Soc. *86*, 759–773.
4. Wolf, M., and Krause, J. (2014). Why personality differences matter for social functioning and social structure. Trends Ecol. Evol. *29*, 306–308.
5. Ward, A.J.W., Thomas, P., Hart, P.J.B., and Krause, J. (2004). Correlates of boldness in three-spined sticklebacks (*Gasterosteus aculeatus*). Behav. Ecol. Sociobiol. *55*, 561–568.
6. Kurvers, R.H.J.M., Eijkelenkamp, B., van Oers, K., van Lith, B., van Wieren, S.E., Ydenberg, R.C., and Prins, H.H.T. (2009). Personality differences explain leadership in barnacle geese. Anim. Behav. *78*, 447–453.
7. Harcourt, J.L., Ang, T.Z., Sweetman, G., Johnstone, R.A., and Manica, A. (2009). Social feedback and the emergence of leaders and followers. Curr. Biol. *19*, 248–252.
8. Pettit, B., Ákos, Z., Vicsek, T., and Biro, D. (2015). Speed determines leadership and leadership determines learning during pigeon flocking. Curr. Biol. *25*, 3132–3137.
9. Pike, T.W., Samanta, M., Lindström, J., and Royle, N.J. (2008). Behavioural phenotype affects social interactions in an animal network. Proc. Biol. Sci. *275*, 2515–2520.
10. Aplin, L.M., Farine, D.R., Morand-Ferron, J., Cole, E.F., Cockburn, A., and Sheldon, B.C. (2013). Individual personalities predict social behaviour in wild networks of great tits (*Parus major*). Ecol. Lett. *16*, 1365–1372.
11. Jolles, J.W., Fleetwood-Wilson, A., Nakayama, S., Stumpe, M.C., Johnstone, R.A., and Manica, A. (2015). The role of social attraction and its link with boldness in the collective movements of three-spined sticklebacks. Anim. Behav. *99*, 147–153.
12. Farine, D.R., Strandburg-Peshkin, A., Couzin, I.D., Berger-Wolf, T.Y., and Crofoot, M.C. (2017). Individual variation in local interaction rules can explain emergent patterns of spatial organization in wild baboons. Proc. Biol. Sci. *284*, 25–29.
13. Dyer, J.R.G., Croft, D.P., Morrell, L.J., and Krause, J. (2009). Shoal composition determines foraging success in the guppy. Behav. Ecol. *20*, 165–171.
14. Laskowski, K.L., Montiglio, P.O., and Pruitt, J.N. (2016). Individual and group performance suffers from social niche disruption. Am. Nat. *187*, 776–785.
15. Pruitt, J.N., and Riechert, S.E. (2011). How within-group behavioural variation and task efficiency enhance fitness in a social group. Proc. Biol. Sci. *278*, 1209–1215.
16. Ioannou, C.C., and Dall, S.R.X. (2016). Individuals that are consistent in risk-taking benefit during collective foraging. Sci. Rep. *6*, 33991.
17. Smith, B.R., and Blumstein, D.T. (2008). Fitness consequences of personality: a meta-analysis. Behav. Ecol. *19*, 448–455.
18. Sih, A., Cote, J., Evans, M., Fogarty, S., and Pruitt, J. (2012). Ecological implications of behavioural syndromes. Ecol. Lett. *15*, 278–289.
19. Réale, D., Dingemanse, N.J., Kazem, A.J.N., and Wright, J. (2010). Evolutionary and ecological approaches to the study of personality. Philos. Trans. R. Soc. Lond. B Biol. Sci. *365*, 3937–3946.
20. Schaerf, T.M., Herbert-Read, J.E., Myerscough, M.R., Sumpter, D.J.T., and Ward, A.J.W. (2016). Identifying differences in the rules of interaction between individuals in moving animal groups. arXiv, arXiv:1601.08202, http://arxiv.org/abs/1601.08202.
21. Couzin, I.D., and Krause, J. (2003). Self-organization and collective behavior in vertebrates. Adv. Stud. Behav. *32*, 1–75.
22. Sumpter, D.J.T. (2010). Collective Animal Behavior (Princeton University Press).
23. Herbert-Read, J.E. (2016). Understanding how animal groups achieve coordinated movement. J. Exp. Biol. *219*, 2971–2983.
24. Couzin, I.D., Krause, J., James, R., Ruxton, G.D., and Franks, N.R. (2002). Collective memory and spatial sorting in animal groups. J. Theor. Biol. *218*, 1–11.
25. Tunstrøm, K., Katz, Y., Ioannou, C.C., Huepe, C., Lutz, M.J., and Couzin, I.D. (2013). Collective states, multistability and transitional behavior in schooling fish. PLoS Comput. Biol. *9*, e1002915.
26. Couzin, I.D., Krause, J., Franks, N.R., and Levin, S.A. (2005). Effective leadership and decision-making in animal groups on the move. Nature *433*, 513–516.
27. Ioannou, C.C., Singh, M., and Couzin, I.D. (2015). Potential leaders trade off goal-oriented and socially oriented behavior in mobile animal groups. Am. Nat. *186*, 284–293.
28. Persson, L., and Eklöv, P. (1995). Prey refuges affecting interactions between piscivorous perch and juvenile perch and roach. Ecology *76*, 70–81.
29. Jolles, J.W., Manica, A., and Boogert, N.J. (2016). Food intake rates of inactive fish are positively linked to boldness in three-spined sticklebacks *Gasterosteus aculeatus*. J. Fish Biol. *88*, 1661–1668.
30. Krause, J., and Ruxton, G.D. (2002). Living in Groups (Oxford University Press).
31. Ward, A., and Webster, M. (2016). Sociality: The Behaviour of Group-Living Animals (Springer International Publishing).
32. Katz, Y., Tunstrøm, K., Ioannou, C.C., Huepe, C., and Couzin, I.D. (2011). Inferring the structure and dynamics of interactions in schooling fish. Proc. Natl. Acad. Sci. USA *108*, 18720–18725.
33. Herbert-Read, J.E., Perna, A., Mann, R.P., Schaerf, T.M., Sumpter, D.J.T., and Ward, A.J.W. (2011). Inferring the rules of interaction of shoaling fish. Proc. Natl. Acad. Sci. USA *108*, 18726–18731.
34. Herbert-Read, J.E., Krause, S., Morrell, L.J., Schaerf, T.M., Krause, J., and Ward, A.J.W. (2012). The role of individuality in collective group movement. Proc. Biol. Sci. *280*, 20122564.
35. Nagy, M., Akos, Z., Biro, D., and Vicsek, T. (2010). Hierarchical group dynamics in pigeon flocks. Nature *464*, 890–893.
36. Johnstone, R.A., and Manica, A. (2011). Evolution of personality differences in leadership. Proc. Natl. Acad. Sci. USA *108*, 8373–8378.
37. Farine, D.R., Montiglio, P.O., and Spiegel, O. (2015). From individuals to groups and back: the evolutionary implications of group phenotypic composition. Trends Ecol. Evol. *30*, 609–621.
38. Spiegel, O., Leu, S.T., Bull, C.M., and Sih, A. (2017). What's your move? Movement as a link between personality and spatial dynamics in animal populations. Ecol. Lett. *20*, 3–18.
39. Borg, B., Bornestaf, C., Hellqvist, A., Schmitz, M., and Mayer, I. (2004). Mechanisms in the photoperiodic control of reproduction in the stickleback. Behav. *141*, 1521–1530.
40. Jolles, J.W., Aaron Taylor, B., and Manica, A. (2016). Recent social conditions affect boldness repeatability in individual sticklebacks. Anim. Behav. *112*, 139–145.
41. Biro, P.A. (2012). Do rapid assays predict repeatability in labile (behavioural) traits? Anim. Behav. *83*, 1295–1300.

42. Webster, M.M., and Laland, K.N. (2009). Evaluation of a non-invasive tagging system for laboratory studies using three-spined sticklebacks *Gasterosteus aculeatus*. J. Fish Biol. *75*, 1868–1873.

43. Wright, D., and Krause, J. (2006). Repeated measures of shoaling tendency in zebrafish (*Danio rerio*) and other small teleost fishes. Nat. Protoc. *1*, 1828–1831.

44. Crawley, M. (2007). The R Book (Wiley).

45. Nakagawa, S., and Schielzeth, H. (2010). Repeatability for Gaussian and non-Gaussian data: a practical guide for biologists. Biol. Rev. Camb. Philos. Soc. *85*, 935–956.

46. Jolles, J.W., Boogert, N.J., Sridhar, V.H., Couzin, I.D., and Manica, A. (2017). Data from: consistent individual differences drive collective behaviour and group functioning of schooling fish (Cambridge University Data Repository). http://dx.doi.org/10.17863/CAM.12136.

STAR★METHODS

KEY RESOURCES TABLE

REAGENT or RESOURCE	SOURCE	IDENTIFIER
Deposited Data		
Raw and analyzed data	This paper	https://doi.org/10.17863/CAM.12136
Experimental Models: Organisms/Strains		
G. aculeatus Wild-type	Local stream near Cambridge, UK	N/A
Software and Algorithms		
Software for running the agent-based models documented in the paper can be found on GitHub	N/A	https://github.com/vivekhsridhar/individual_differences

CONTACT FOR REAGENT AND RESOURCE SHARING

Further information and requests for resources and reagents should be directed to and will be fulfilled by the Lead Contact, Jolle W. Jolles (j.w.jolles@gmail.com).

EXPERIMENTAL MODEL AND SUBJECT DETAILS

We collected three-spined sticklebacks (*Gasterosteus aculeatus*) during the summer of 2014 from a stream near Cambridge, England, and housed them in our lab under controlled temperature (14°C ± 1°C) and light (12 hr:12 hr light:dark) conditions. Fish were kept in large glass tanks (120 cm length × 60 cm width × 60 cm height) with artificial plants and shelters, which were maintained by both under-gravel and external filtration. Fish were fed defrosted bloodworms (*Chironomid* larvae) ad libitum once daily. After an acclimatization period of six months, when fish were about nine months old, we randomly selected 125 individuals, controlling for size (body length 'BL' ± SE: 40.6 ± 0.4 mm), and moved them to individual compartments (18.5 cm × 9.5 cm), each lined with gravel and containing an artificial plant, where they were kept for the remainder of the experiment. Compartments were divided from neighboring compartments by perforated transparent partitions. We pseudo-randomly (controlling for holding tank to minimize potential familiarity effects) allocated individuals to one of 25 groups of five after the completion of the individual behavioral assays (described below). Since it is impossible to non-invasively sex sticklebacks outside the breeding season, all groups were assumed to be of mixed sex, with group sex ratio unlikely to have a big impact on our results under these controlled laboratory conditions [39] as both sexes are non-territorial and actively shoal together. During the whole experimental period, fish were fed three bloodworms at the end of each day. Animal care and experimental procedures were approved by the Animal Users Management Committee of the University of Cambridge as a non-regulated procedures-regime.

METHOD DETAILS

Experimental overview

To control for potential social modulation and acclimatization effects [40, 41], experiments started three days after individual housing. We started with the individual behavioral assays and subjected fish to a classic 'boldness' assay on experimental days 4 and 8 and a classic 'sociability' assay on days 6 and 10. We then allocated individuals to groups of five, which is a common group size for stream-inhabiting sticklebacks and conforms with previous work, which has predominantly looked at group sizes between 2-30 individuals [7, 13, 32, 33]. Group size and composition were kept constant throughout the experimental period. To enable individual identification in the groups, after two rest days (day 13) we tagged fish on their middle dorsal spine with a uniquely colored disc-shaped tag (6 mm diameter) made from colored electrical tape. This non-invasive tagging method only took between 15-30 s per fish and has been shown to have no major effects on either the activity or shoaling behavior of three-spined sticklebacks [42]. After another rest day, we started with the shoaling experiments using two replicates of a large circular tank. The experimenters were blind to the identity of the fish and the composition of the groups. On day 15 we tested groups in the open tanks without food or cover, on days 16 and 17, twice per day, with patches of food but without plant cover, and on days 18 and 19 with food patches and a plant cover.

Individual behavioral assays

Individual fish (n = 125) were tested using two standard personality assays, conventionally used to quantify boldness and sociability [7, 16, 43]. The asocial boldness assay consisted of a white Perspex tank (55 cm × 15 cm × 20 cm) containing a deep area (15 cm × 10 cm; 13 cm depth) with an artificial plant as refuge, and an open sandy area with a slope leading to shallow water (3 cm) at the other side (Figure S1A). The social assay consisted of a tank (50 cm × 30 cm, 8 cm depth) that was lengthwise divided by two transparent partitions to create one larger middle compartment (30 cm width), used for the focal fish, and two smaller side compartments

(10 cm width), one of which contained five conspecifics (Figure S1E). At the start of each test day the fish forming the conspecifics shoal were randomly selected from the stock tanks and allowed to acclimatize to the compartment for 45 min. The position of the compartment housing the five fish was then randomly selected every four trials after which the shoal was allowed to acclimatize for 10 more minutes before the start of the next trial. We calculated an 'exploratory' and 'social proximity' score for each fish by respectively averaging the proportion of time fish spent out of cover and averaging their mean distance from the shoal compartment across the two trials of each assay. For both assays we also measured fish's swim speed as a function of their distance out of cover/from the shoal compartment (see Figure S1). Trials lasted 30 and 15 min for the asocial boldness assay and the social assay respectively. For both assays, fish were taken from their individual compartment at the start of a trial and returned there immediately after completing the trial using a dip net. We used a custom replicated set-up of eight boxes that enabled us to test multiple fish simultaneously under identical conditions, while minimizing outside disturbances. Sessions were automatically recorded at 12 fps in high-definition using Raspberry Pi computers (Raspberry Pi Foundation, England) positioned in the top of each box.

Group shoaling experiments

To investigate the collective behavior of the fish, groups were repeatedly subjected to a white, circular Perspex tank (80 cm diameter, 20 cm height; 7 cm water depth), positioned inside a large white light tent (200 cm × 100 cm × 160 cm) illuminated from the top and sides. For the fish, the tank is a potentially dangerous environment due to being bright, open, and homogeneous, and results in fish to strongly school together (see Figure S2). For the trials in the foraging contexts we placed three food patches at random locations in roughly equilateral triangular formation in the tank, between 5 cm from the wall and 15 cm from the tank center. Food patches consisted of white Perspex grids (5 cm × 5 cm × 1 cm) containing five bloodworms each, randomly distributed among the grids' 16 cells. The patches were constructed such that fish would notice the prey items from a distance of approximately 10-15 cm. For the trials in the semi-covered foraging context, artificial plants were positioned in the center of the tank, creating a covered area with a diameter of 15 cm.

Each group received a total of seven test trials: one in the classic context (30 min), four in the open foraging context (5 min), and two in the foraging plus cover context (10 min). The group order of testing was randomized but a fixed context order was used as not to confound the behavior of the fish in the earlier contexts with experience of the foraging patches and cover being available. Data analysis of the free-schooling context trials focused on the first 5 min only (c.f. [33]) but trials lasted 30 min to enable the analysis of certain temporal effects (see below). Before each trial, fish were taken from their individual compartment using a dip net and allowed to acclimatize for 30 s in black plastic cups, after which all five fish of a group were simultaneously placed in a transparent Perspex cylinder (10 cm diameter) in the center of the tank. After another 30 s acclimatization, the fish were released by remotely raising the cylinder. At the end of each trial, fish were placed back in their compartments, any fish droppings and remaining food items removed, and tank water circulated to mix any chemical cues. Trials were recorded from above at 24 fps at a resolution of 1400 × 1400 using Raspberry Pi computers. As groups received two foraging trials per day, with five bloodworms provided in each foraging patch, hypothetically a fish could reach a maximum daily food intake of 30 food items. This was by far never observed. Furthermore, sticklebacks under similar conditions are capable of consuming up to 60 bloodworms within a three-hour time span [29]. Satiation is therefore unlikely to have had a strong effect on the observed foraging performance.

Automated tracking and data collection

We acquired highly detailed individual-based movement data for both the individual and group assays with custom tracking software written in Python version 2.7.12 (AnimTrack, by J.W.J.) based on the OpenCV library. For the individual trials, a background image, created by averaging the first 200 frames, was subtracted from each frame and fish were subsequently identified via automatic thresholding using constant threshold values. For the group trials we automatically identified fish based on their differently colored tags, which enabled us to acquire highly accurate tracking data linked to each individual, despite occasional occlusions. Positional coordinates were converted from pixels to mm and subsequently smoothed using a Savitzky & Sgolay smoothing filter with a window of 15 frames. After tracking, all trajectory data were visually checked for any inconsistencies or errors and, if needed, manually corrected. In addition, we performed manual video observations for the trials in the foraging contexts and recorded the time each food item was eaten (0.1 s precision) as well as the identity of the foraging fish.

Individual-based modeling

Overview

We adapted the simple spatially-explicit self-propelled particle model detailed in Couzin et al. [24] and combined it with goal-oriented behavior (omega) [26, 27], which has been shown to be an important factor in individuals' responses to known resource locations. We deliberately chose this simple model, not to obtain a quantitative comparison to the experiments, but to determine if the general results are consistent with theory, and to seek a parsimonious explanation for the observed patterns.

Framework

Groups were composed of individuals, each characterized by a position vector $c_i(t)$, a unit direction vector $\widehat{v_1}(t)$ and speed $|v_i(t)|$, where i is the identity of the individual and t is the current time step. The speed of each individual is drawn from a normal distribution to represent consistent inter-individual differences. Hence, each individual differs in speed and a given individual's speed remains constant within a simulation. While having a constant speed is an oversimplification (to obtain the simplest possible model formulation that can explain the experimental results), due to the nature of response to social interactions, individuals can effectively slow down, or speed up, by virtue of modifications to the small-scale tortuosity of their motion. For example, fast individuals at the front of groups

will tend to be attracted to those behind, resulting in them taking a more tortuous path, effectively slowing them in the direction of travel of the group as a whole, whereas slower individuals trailing groups will exhibit highly directed motion that increases their relative speed in the direction of travel with respect to other group members (see Movie S3).

Social interactions with others were accounted for through three types of interactions: repulsion, alignment and attraction. Individuals turn away from n_r neighbors encountered within a small radius (r_r) around them. This represents collision avoidance and maintenance of personal space expressed by the agents, and, as is apparent in real schools [30, 31], takes highest priority.

$$s_r(t) = -\sum_{j \neq i}^{n_r} \frac{c_j(t) - c_i(t)}{|c_j(t) - c_i(t)|} \quad \text{(Equation 1)}$$

where $s_r(t)$ represents the social component of an individual's desired direction of motion after responding to individuals within r_r.

If no individual is present within radius r_r, the focal individual orients itself with individuals within r_o and is attracted to individuals in zone r_a These zones are circular, with a blind area of a° behind the individual. In these zones, individuals interact with conspecifics only in the remaining $(360 - a)^\circ$. All three zones are non-overlapping and their widths are defined as $\Delta r_r = r_r$, $\Delta r_o = r_o - r_r$, and $\Delta r_a = r_a - r_o$. Since we simulated a group of five individuals, and due to the relatively small environment in which experiments were conducted where individuals can readily see others at the maximum possible spacing, we set the maximal range of perception r_a to ∞. Each individual attempts to align its direction of motion with n_o neighbors in the zone of orientation, giving

$$s_o(t) = -\sum_{j=i}^{n_o} \frac{v_j(t)}{|v_j(t)|} \quad \text{(Equation 2)}$$

and is attracted toward positions of individuals within the zone of attraction

$$s_a(t) = -\sum_{j \neq i}^{n_a} \frac{c_j(t) - c_i(t)}{|c_j(t) - c_i(t)|}. \quad \text{(Equation 3)}$$

Once individuals have a social vector, they reconcile this with their goal-oriented tendency $g_i(t)$ weighted by a continuous term ω, which represents the strength of individual goal-orientedness. Like speed, individual ω is drawn from a Gaussian distribution (to represent consistent inter-individual differences) and remains constant in a given simulation

$$d_i(t + \Delta t) = \sum_{i=1}^{n} \frac{s_i(t)}{|s_i(t)|} + \omega \widehat{g}_i(t). \quad \text{(Equation 4)}$$

$d_i(t+\Delta t)$ is then normalized $\widehat{d}_i(t+\Delta t) = d_i(t+\Delta t)/|d_i(t+\Delta t)|$, to represent the desired direction of motion of the individual. Individuals' goal-oriented vector $g_i(t)$ points in the direction of their current motion until they enter a radius r_c of a rewarding cue. This can be interpreted as their inertia, or their desire to continue moving in their current direction when reward is not perceived. Once individuals are within this set radius, their goal-oriented vector $g_i(t)$ is directed toward the reward to an extent determined by their ω. Once individuals are on a food patch, they feed with a feeding rate f.

Motion of all individuals is subject to noise (error in movement and/or sensory integration) which is implemented by rotating $\widehat{d}_i(t+\Delta t)$ by a random angle chosen from a circularly wrapped Gaussian distribution centered at 0 and of standard deviation e. Once the desired direction has been determined, individuals turn toward $\widehat{d}_i(t+\Delta t)$ with a maximum turning rate of $\psi \Delta t$.

In the foraging context (see below), boundary conditions were enforced by modifying the desired direction of an individual to equal a boundary vector $b_i(t)$ when they reached a narrow zone near the edge of the arena. Boundary vector $b_i(t)$ is a unit vector pointing toward the center of the arena. This was done to allow agents to avoid walls and to prevent them from leaving the arena. In the free schooling context, individuals were initialized in a periodic boundary environment to ensure that no boundary related artifacts are observed while measuring spatial positioning of individuals.

An overview of the parameters used in the individual-based models, with asterisks indicating parameters for the foraging context only, can be found in the below table.

Parameter	Symbol	Values explored
Arena size	A	500
Zone of repulsion	r_r	1
Zone of orientation	r_o	6
Zone of attraction	r_a	∞
Field of perception	α	270°
Turning rate	ψ	60°
Speed	$\|v\|$	0.1–2.0
Speed error	e_s	0.1

(*Continued on next page*)

Continued		
Parameter	Symbol	Values explored
Omega	ω	0.01–0.1
Omega error	e_ω	0.01
Timestep increment	Δ_t	0.1
Cue detection radius	r_c	30
Nr of food patches*		3
Nr of food items per patch*		50
Feeding rate*	f	0.001

Simulations

In line with the experiments, we started with simulations of groups composed of five individuals. To simulate the free-schooling context and open foraging context presented in the experiments, we initialized the groups both in an open, boundary free environment and in a circular environment that contained three food patches (10 units radius). Individuals were initialized with random positions and directions in the middle of the arena, again in line with the experimental procedure. Details about model parametrization can be found in the above table. Parameter values for the schooling models are standard values, previously explored in [24, 25]. To explore further how the effects may be group-size dependent, we ran additional simulations with larger groups of twenty. As speed distributions are often right-skewed and bound at zero, including our experimental data (skew: 0.289; see Figure S1I), we also ran simulations of the free-schooling context (for one specific parameter condition) with a Gamma distribution of shape parameter ($k = 0.4$ and scale parameter, $\theta = \sqrt{0.05}$). These parameters were chosen so that the distribution had a mean within our tested range and variance identical to the one used in case of the Gaussian distribution. For the free-schooling context we ran simulations of 2,000 time steps and for the foraging context 10,000, with data being stored every 200 and 500 time steps respectively, with 400 replicates of each parameter condition explored.

QUANTIFICATION AND STATISTICAL ANALYSIS

Computation of behavioral data

Individual characteristics

We determined each fish's velocity, speed, direction, acceleration, and turning speed directly from the discrete tracking data using the following series of calculations. With the vector $\mathbf{r}_i(t) = (x_i(t), y_i(t))$ denoting the position of fish i at time t, we approximated its velocity $\mathbf{v}_i(t) = (u_i(t), w_i(t))$ using the forward finite difference

$$\mathbf{v}_i(t) = \frac{\mathbf{r}_i(t + \Delta t) - \mathbf{r}_i(t)}{\Delta t}, \quad \text{(Equation 5)}$$

where $\Delta t = 1/24$ s is the time interval between subsequent position measurements. The speed $v_i(t)$ is then given by the norm of the velocity vector, such that

$$v_i(t) = |\mathbf{v}_i(t)| = \sqrt{u_i^2(t) + w_i^2(t)}. \quad \text{(Equation 6)}$$

Next, we quantified the direction of motion using the angle $\psi_i(t)$ between the velocity vector and the positie y axis, which is given by

$$\psi_i(t) = \text{atan2}(w_i(t),\ u_i(t)) \quad \text{(Equation 7)}$$

Furthermore, we quantified the acceleration as a finite difference of the velocity

$$\mathbf{a}_i(t) = \frac{\mathbf{r}_i(t + \Delta t) - 2\mathbf{r}_i(t) + \mathbf{r}_i(t - \Delta t)}{\Delta t^2}, \quad \text{(Equation 8)}$$

and the turning speed, or angular velocity, as a finite difference of the angle,

$$\gamma_i(t) = \frac{\psi_i(t + \Delta t) - \psi_i(t)}{\Delta t}. \quad \text{(Equation 9)}$$

As fish were placed at the origin of the Cartesian coordinate system pointing north, care was taken to compute the correct angular difference with regard to the periodicity of $\psi_i(t)$, anti-clockwise from 0° to −180° and clockwise from 0° to 180°.

$$\{\gamma_i(t) < -\pi)\} : \gamma_i = 2\pi - |\gamma_i(t)| \quad \text{or} \quad \{\gamma_i(t) > \pi)\} : -(2\pi - \gamma_i(t)). \quad \text{(Equation 10)}$$

Within group positioning

We determined the positioning and ordering of the fish in a group relative to one another and to the direction of motion of the group center using the following calculations and linear transformations. To calculate fish nearest neighbor distance (NND), we computed a matrix of distances between all individuals and then determined the minimum value for each fish such that

$$\mathrm{NND}_i(t) = min_{j \neq i}\left(\sqrt{(x_i(t) - x_j(t))^2 + \left(y_i(t) - y_j(t)\right)^2}\right), \qquad \text{(Equation 11)}$$

where j indexes all neighbors of fish i.

Next, for each time step we identified the mean coordinates of all fish in a group $\mathbf{r_c}(t) = (x_c(t), y_c(t))$, that is, the group center, and then estimated the velocity $v_c(t)$ and direction $\psi_c(t)$ of the group center at time t using the calculations as for the individual fish (described above). Then for each frame we calculated the distance of each fish to the group center as

$$\mathrm{CD}_i(t) = \sqrt{(x_i(t) - x_c(t))^2 + (y_i(t) - y_c(t))^2}. \qquad \text{(Equation 12)}$$

To calculate relative positions of individuals to the group, we shifted the coordinates of each fish so that the origin of the coordinate system was at the group centroid, and determined the angle between the positive y axis through the group centroid and an individual's position

$$\delta_i(t) = \mathrm{atan2}(x_i(t) - x_c(t), y_i(t) - y_c(t)). \qquad \text{(Equation 13)}$$

Subsequently, we used this to calculate an individual's relative direction to that of the group center

$$\sigma_t(t) = \delta_i(t) - \psi_c(t), \qquad \text{(Equation 14)}$$

which we then adapted to fit to the Cartesian coordinate system pointing north

$$\{\sigma_t(t) < -\pi)\} : \sigma_t = 2\pi - |\sigma_t(t)| \quad \text{or} \quad \{\sigma_t(t) > \pi)\} : -(2\pi - \sigma_i(t)) \qquad \text{(Equation 15)}$$

Based on the relative direction and distance to the group center, we calculated the relative position for each fish to the group center:

$$\left(x_i', y_i'\right) = \mathrm{CD}_i(t)(\sin(\sigma_i(t)), \cos(s_i(t))). \qquad \text{(Equation 16)}$$

The transformed coordinates of the fish meant that fish with greater y-coordinates were at the front for a given time step. We then counted the proportion of frames that each fish was located in front of the group center. To further examine inter-individual positioning in the group, we calculated fish's relative direction to that of its four group mates θ_{ij} from the respective angles of the fish with the y axis (ψ_j) following the calculations as used for the relative positioning to the group center.

Group characteristics

To examine the properties of the differently composed groups, we calculated the speed of the group center, group cohesion, and polarization using the following calculations. For each time step t, the speed of the group $v_c(t)$ is given by the norm of the velocity vector, such that

$$v_c(t) = |\mathbf{v}_c(t)|. \qquad \text{(Equation 17)}$$

We then calculated the mean inter-individual distance $\mathrm{IID}_c(t)$ as a measure of group cohesion, based on the individual distances IID_{ij} between all fish (n) in a group

$$\mathrm{IID}_c(t) = \frac{1}{n}\sum_{j \neq i}^{n} \mathrm{IID}_{ij} \qquad \text{(Equation 18)}$$

using

$$\mathrm{IID}_{ij} = \sqrt{(x_i(t) - x_j(t))^2 + \left(y_i(t) - y_j(t)\right)^2}. \qquad \text{(Equation 19)}$$

And finally we calculated the polarization of the group

$$\rho(t) = \frac{1}{n}\sqrt{\left(\sum_{i=1}^{n} \sin(\psi_i(t))\right)^2 + \left(\sum_{i=1}^{n} \cos(\psi_i(t))\right)^2}, \qquad \text{(Equation 20)}$$

which is a measure of the alignment of the fish in the group relative to each other, and ranges from 0 (complete non-alignment) to 1 (complete alignment).

Schooling is defined as a cohesive group that moves with considerable speed and alignment, while a group is said to swarm when it is cohesive but has no or little speed and/or alignment between its members [21]. To investigate the schooling tendency of the groups, we computed the distributions of the three fundamental components of schooling for the full dataset: group cohesion, speed, and polarization (see Figure 3 and Figure S2). Furthermore, based on the detailed distributions of all groups as well as parameters

from previous work [25, 32], for each frame we also categorized groups to school, based on the following criteria: mean inter-individual distance IID $\leq$ 160 mm, speed of group center vector $v_c \geq 0.5$ BL/s, polarization $\rho \geq 0.6$, no outliers or group split. Outliers and group splits were computationally identified based on a non-linear distribution of ordered distances between all group members in terms of the IID and NND, with parameters identified based on the raw data distributions Those frames in which outliers or group splits occurred were scored as 'non-schooling.' To check the robustness of the schooling measure and selected parameter combination, we checked 124 alternative parameter combinations with Spearman rank correlations: group polarization (0.4–0.8 with 0.1 increments), speed (1.0–3.0 cm/s with 0.5 cm/s increments), and cohesion (iid 100–220 mm with 30 mm increments) and found that over 80% of these combinations were significant and 93% showed a trend for an effect with group social proximity tendency (see main text).

Propagation of motion

To investigate leadership in terms of the propagation of movement changes in the group, we examined temporal correlations in speeding and turning changes for all dyads within all groups [32, 35]. We compared the speed and direction of the two fish in a dyad up to 72 frames (3 s) earlier and later, in time steps of $1/24^{th}$ s, and quantified the mean time point of the maximum correlation coefficient (see Figure S3). A leading event was said to have occurred when a fish's change in speed or direction was 'copied' by another fish delayed in time. Subsequently, we constructed leadership networks based on the time delays between all group members following Nagy et al. [35]. Analysis was restricted to frames in which fish were less than four BL apart and moved faster than 1 BL/s.

Foraging and hiding behavior

For the trials in the two foraging contexts we used the positional data to compute the order that individuals arrived in the vicinity ($\leq$30 mm) of and above the foraging patches. We defined the first fish to 'discover' a foraging patch as the one that first arrived in its vicinity during a trial. For the trials in the semi-covered foraging context we also calculated the proportion of time individuals spent out of cover (with at least half their body), the proportion of time individuals spent out of cover alone, and their mean order number for leaving cover. In turn, these measures were used to calculate the mean number of fish out of cover and the proportion of time all fish were out of cover.

Data analysis

Data were analyzed in R 3.2.0. We used a generalized linear mixed modeling (GLMM) approach [44] to investigate the effects of inter-individual behavioral differences on behavioral repeatability as well as individual and group shoaling and foraging behavior. To assess individual behavioral consistency, we calculated Consistency Repeatability [45] using linear mixed models that included day as a fixed effect and fish ID as a random factor. We calculated 95% confidence intervals of repeatability by running 10,000 permutations of each test. Significant effects are those with a confidence interval that does not overlap 0. Exploration and social proximity scores were scaled between 0 and 1, with social proximity values square-root transformed and inverted before scaling, with higher scores indicating a stronger tendency. To compute relative scores, we calculated the mean behavioral score of a fish's group mates and subtracted that from the focal fish's behavioral score. Neither fish's exploratory tendency nor their social proximity tendency was significantly correlated with body size (Pearson correlation test: $r_{123} = 0.02$, p = 0.804 and $r_{123} = -0.03$, p = 0.759). The randomized group compositions (n = 25 groups) were normally distributed in terms of the mean personality types.

For the behaviors in the free-shoaling experiments, response variables were calculated based on the distribution of the data on a frame-by-frame basis, with mean values calculated for approximately normal (transformed) distributions and median values when data was skewed. For the individual-level models we included individual exploration and social proximity scores and the interaction between them as fixed effects. Group identity was fitted as a random factor to account for the non-independence of individuals within a group, and individual identity nested in group identity was additionally included for the trials in the two foraging contexts to account for the repeated-measures-nature of the data. For the group-level models we fitted the mean exploratory and mean social proximity tendency of the group and the interaction between them. We only included measures of group variability in behavioral tendencies in the case of clear a priori hypotheses as not to over-parametrise our models. Food intake and the likelihood to discover the foraging patches were fitted to a Poisson error distribution with log link function, appropriate for count data. To investigate how the effect of inter-individual differences on the proportion of time fish spent in the front of the group changed over time, we compared models based on the first 5 min and all 30 min of the trial. To investigate the propagation of speeding and turning changes in the groups, we ran an ordinal logistic regression with individual exploratory and social proximity tendency ranks in the group as fixed factors, and the random data structure as described above. We analyzed the foraging behavior of individual fish and the groups over time with Cox proportional hazards (survival) regression models. Survival analyses avoid censoring the data, thereby allowing for the assumption that fish or groups assigned to maximum time may have foraged or finished all the food respectively had the trials run longer. For these analyses, the data were clustered around fish identity and group identity to account for dependence in the data and for trial to account for changes over time.

Minimal adequate models were obtained by backward stepwise elimination following Crawley [44], i.e., sequentially dropping the least significant terms from the full model, until all terms in the model were significant (all interaction terms were non-significant unless documented). Statistics for non-significant terms were obtained by adding the term to the minimal model. We also report ΔAIC when comparing models when based on different subsets of the data. Residuals were visually inspected to ensure homogeneity of variance, normality of error and linearity where appropriate. Differences in variance were analyzed using a Levene's test, making sure there was no difference in variance in the personality composition of those groups. Data were log- or square-root transformed

if assumptions were violated, or, where appropriate, a robust Spearman rank correlation test was used. We initially also incorporated body size as covariate in our models, but these effects were non-significant ($p > 0.25$, results not reported) and were consequently removed from the models before refitting. We had to exclude one group onward from the 4th open foraging context trial due to the death of one fish, and one trial in the open foraging context and one trial in the semi-covered foraging context due to experimental errors. For two trials in the semi-covered foraging context no foraging data could be collected due to a recording error. One group was excluded from spatial positioning analysis in the free-schooling context due to an extreme outlier (8.6 SD > mean), which did not qualitatively affect the results. To control for multiple testing, we employed a False Discovery Rate (FDR) correction for all statistical tests using the build-in function in R (stats package). FDR is an alternative, relatively powerful method compared to family-wise error procedures to control for type I errors. Corrected p values are stated in the text. A table with the uncorrected p values can be found in the deposited dataset online. $p < 0.05$ is reported as significant and means are quoted ±SEM throughout unless stated otherwise. Other statistical parameters are reported in the main text and figure legends.

DATA AND SOFTWARE AVAILABILITY

Datasets from the experiments and individual-based modeling are deposited at the University of Cambridge data depository (https://doi.org/10.17863/CAM.12136) [46].

Current Biology, Volume *27*

Supplemental Information

Consistent Individual Differences Drive Collective Behavior and Group Functioning of Schooling Fish

Jolle W. Jolles, Neeltje J. Boogert, Vivek H. Sridhar, Iain D. Couzin, and Andrea Manica

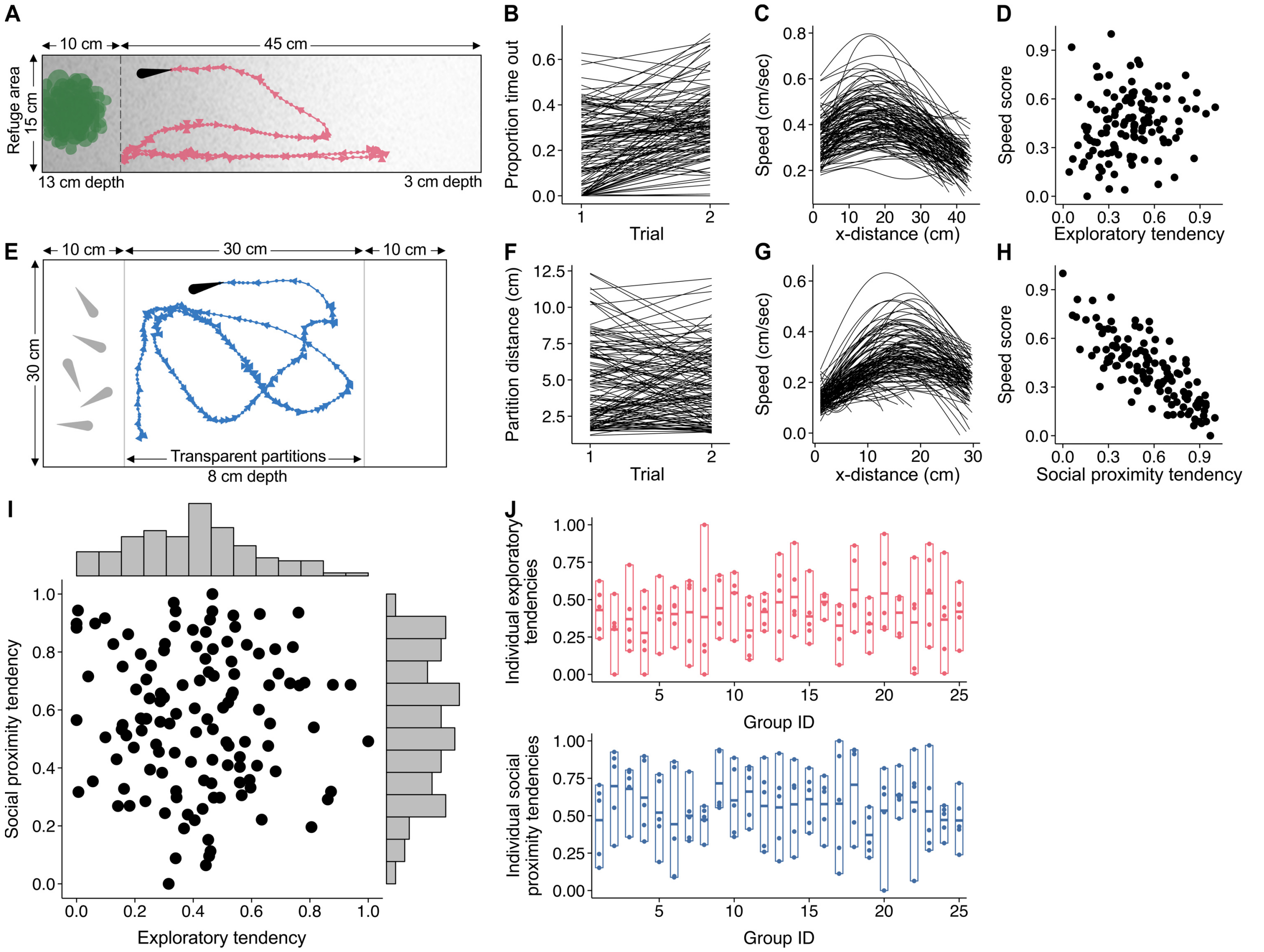

Figure S1. Consistent inter-individual behavioural differences. Related to Figure 1. (**A**) Schematic of the asocial boldness assay, a rectangular tank with a deep refuge area that leads to an increasingly shallow open area on the other side. (**B**) Line plot showing individual repeatability in terms of the proportion of time fish spent out of the refuge ('exploratory tendency'), which was strongly, positively linked to their average distance out of cover ($r_{123} = 0.67, \mathrm{p} < 0.001$). **C**) Line plot showing predicted speed curves (quadratic fit) for both boldness test trials of all fish in terms of their distance out of cover. These speed curves were used to calculate a speed score for each fish by determining the speed where the curve was maximal, averaged across both trials. **D**) Relationship between fish's exploratory tendency and their speed score (scaled) in the boldness assay ($r_{118} = 0.17, \mathrm{p} = 0.104$). (**E**) Schematic of the social assay, a tank with a large centre compartment for the focal fish, and two side compartments, one empty and one containing five conspecifics. (**F**) Line plot showing individual repeatability in terms of the average distance from the conspecifics' compartment ('social proximity tendency'). (**G**) Line plot showing predicted speed curves (quadratic fit) for both test trials of all fish in the social assay in terms of their distance from the compartment holding the shoal. (**H**) Relationship between fish's social proximity tendency and their speed score (scaled) in the social assay ($r_{123} = -0.79, \mathrm{p} < 0.001$). (**I**) Relationship between the exploratory and social proximity tendencies (n = 125 fish) and their distributions (grey bars), with behavioural scores scaled between 0 and 1. (**J**) Group compositions in terms of the individual group members' exploratory and social proximity tendencies (n = 25 groups of 5). Together, these plots show that fish were highly repeatable in their tendency to explore out of cover, as well as in their propensity to stay near the confined shoal in the sociability assay, and that no link existed between them. Fish swam faster the further they were out of cover and the further they were away from the conspecifics' compartment, towards the middle of the tank used in the two assays. While fish's exploratory tendency was only weakly linked to swim speed (**D**), fish's social proximity tendency was strongly negatively linked with swim speed (**H**), even when speed was measured in the asocial boldness assay ($r_{118} = -0.27, \mathrm{p} = 0.008$). Fish were consistent in their swim speed (speed scores) between the trials of the asocial boldness assay ($R_C = 0.41, 0.24 - 0.56$), between the trials of the social assay ($R_C = 0.58, 0.56 - 0.68$), and between the two assays ($R_C = 0.44, 0.29 - 0.56$).

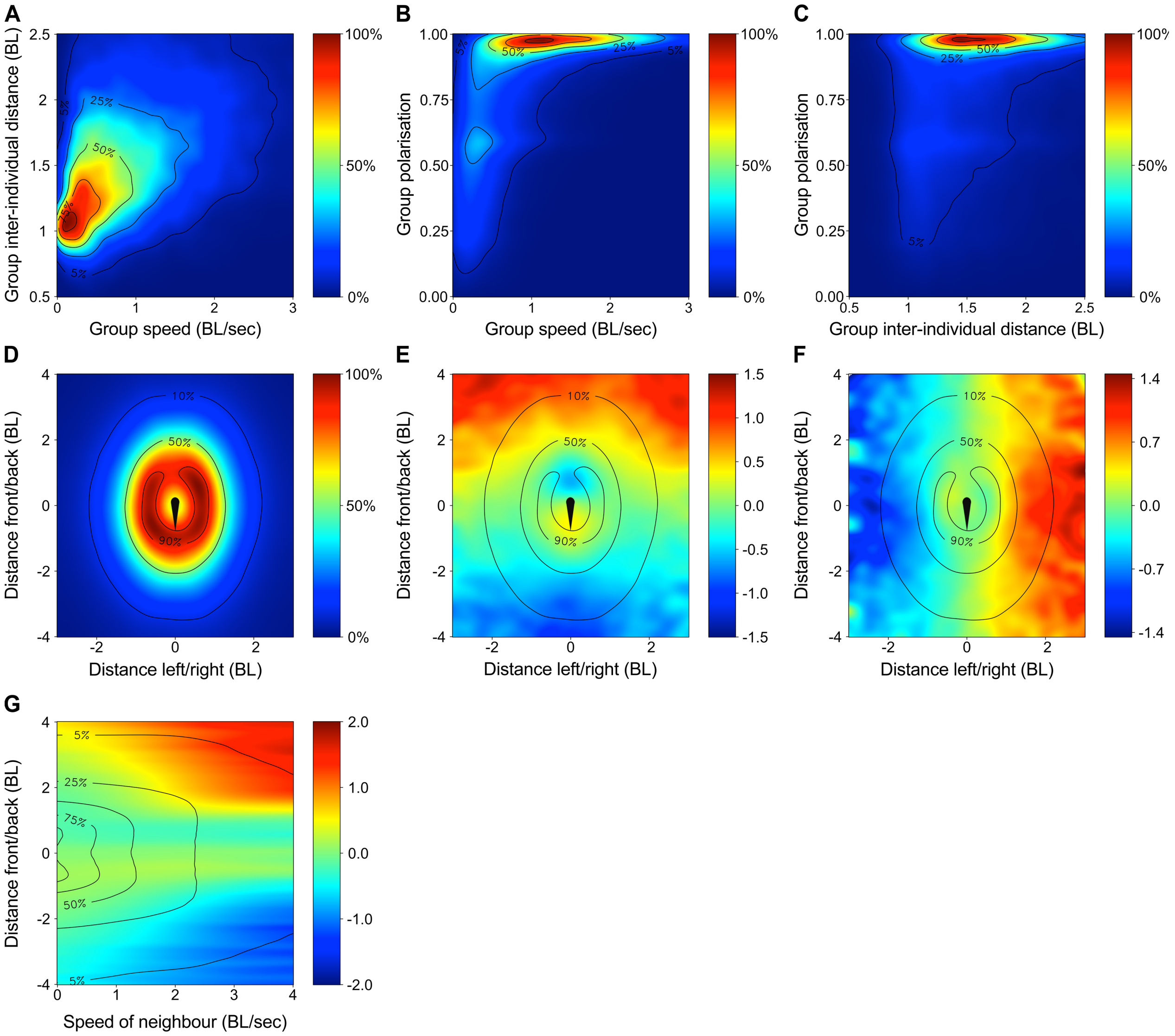

Figure S2. Heat maps of group and individual movement dynamics in the free-schooling context. Related to Figure 2 and 3. (**A**) Relationship between group speed and cohesion, (**B**) group speed and polarisation, and (**C**) group cohesion and polarisation, with group speed depicting the average median speed of the individuals in a group. Measures are expressed in units average body length (BL; 40.6 mm) where appropriate. Plots are based on the frame-by-frame data at time steps of 1/24th sec, with data cropped to show the most relevant area only (respectively 86.9%, 88.9%, and 97.5% of the full parameter space). Contours represent iso-levels in percentage of the highest bin for data of all groups combined. These plots indicate a strong link between group cohesion, speed, and polarisation, with faster moving groups being less cohesive and more strongly aligned. Groups moved at a steady median pace of 30.0 mm/sec, with an average group cohesion ($\overline{\text{IID}}$) of 70.7 mm. In the direction of motion, groups had an average length of 100 mm and rarely exceeded 300 mm. Groups were strongly polarised the majority of the time (median = 0.92) and had very low levels of fragmentation, with significant outliers or group splits (for explanation, see Methods) only occurring 4.9 ± 1.5% of the time. (**D-G**) To investigate the individual interaction rules, we selected each fish in each group and computed its position, acceleration, and turning forces relative to the position and speed of its group mates (see Methods). (**D**) The probability of finding neighbouring fish at a given position relative to the position of the focal fish, which was placed at the origin pointing north. Fish density is presented in percentages relative to the densest bin for all groups combined. (**E**) and (**F**) Respectively the acceleration and turning speed of the focal fish as a function of the position of its group mates. (**G**) Focal fish's acceleration forces as a function of the swim speed of its group mates and its front-back distance. For the turning speed, positive values indicate a right turn and negative values a left turn. Data was based on the full 30 min trial but cropped to show the most relevant area only (**D-F**: 92.1% and **G** 93.3% of the full parameter space). These plots indicate that on average, (**D**) fish are very likely to be within one body length of another group member side-by-side, and within two body lengths front-to-back ($\overline{\text{NND}}$ = 39.0 mm), (**E**) fish speed up when a neighbouring fish is far ahead or just behind them, but slow down when a neighbouring fish is far behind or just in front, (**F**) fish turn left when a neighbouring fish is on its far left side and turn right when its neighbour is on its far right side, with weaker opposite turning tendencies when neighbouring fish are very close, and (**G**) fish acceleration forces become stronger the faster the neighbouring fish is moving.

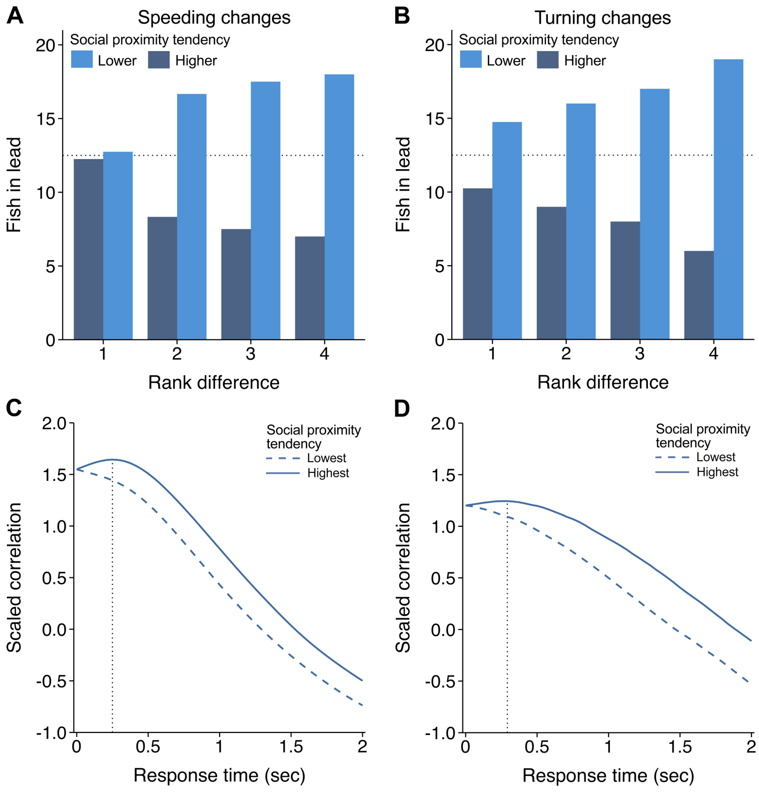

Figure S3. Propagation of movement changes in the free-schooling context. Related to Figure 2. To investigate the propagation of movement changes, we selected each fish in each group as focal individual and compared its swim speed and direction to that of all its group mates up to three seconds later, at time steps of 1/24th sec. We then determined the average time difference for the highest correlation across the trial for all dyads in each group. Fish were ranked based on their social proximity tendency (rank 1-5) within each group. (**A**) and (**B**) Bar plots depicting number of dyads for which movement changes on average propagated from the fish with the higher social proximity tendency versus from the fish with the lower social proximity tendency. Bars show mean values for rank difference of 1-3 (n = 100, 75, 50 respectively) and total number for a rank difference of 4. Dotted line represents the value that both personality ranks would lead equally. (**C**) and (**D**) Median correlations in movement changes for the fish with the highest social proximity tendency relative to fish with the lowest social proximity tendency in each group and the other way around. Correlation coefficients were scaled for each group to control for between-group variability, and analysis was restricted to frames in which both fish were moving at a speed of at least 10 mm/sec during the full 30 min trial in the free-schooling context. Both the (**C**) swim speed correlation and the (**D**) turning correlation of fish with the highest social proximity tendency in a group peaked after zero with a delay time of less than 0.5 sec before decaying (indicated by the grey dotted line), whereas for fish with the lowest social proximity tendency the correlation curve does not show such a peak. This suggests that fish with a higher social proximity tendency on average speed up, slow down and turn in response to the speed and direction of fish that have a relatively lower social proximity tendency. Both speeding, $r_{123} = 0.65, \mathrm{p} < 0.001$, and turning changes, $r_{123} = 0.54, \mathrm{p} < 0.001$, were positively linked with the tendency to be in front. These plots thus show that fish with a higher relative tendency for social proximity, which moved faster in the solitary and group assays and were more in front, are more likely to lead their group mates in terms of both (**A**) the propagation of speeding changes (ordered logistic regression: $z = -2.78, \mathrm{p} = 0.012$) and (**B**) the propagation of turning changes ($z = -2.76, \mathrm{p} = 0.012$), and that this increases the larger the rank difference between the two fish.

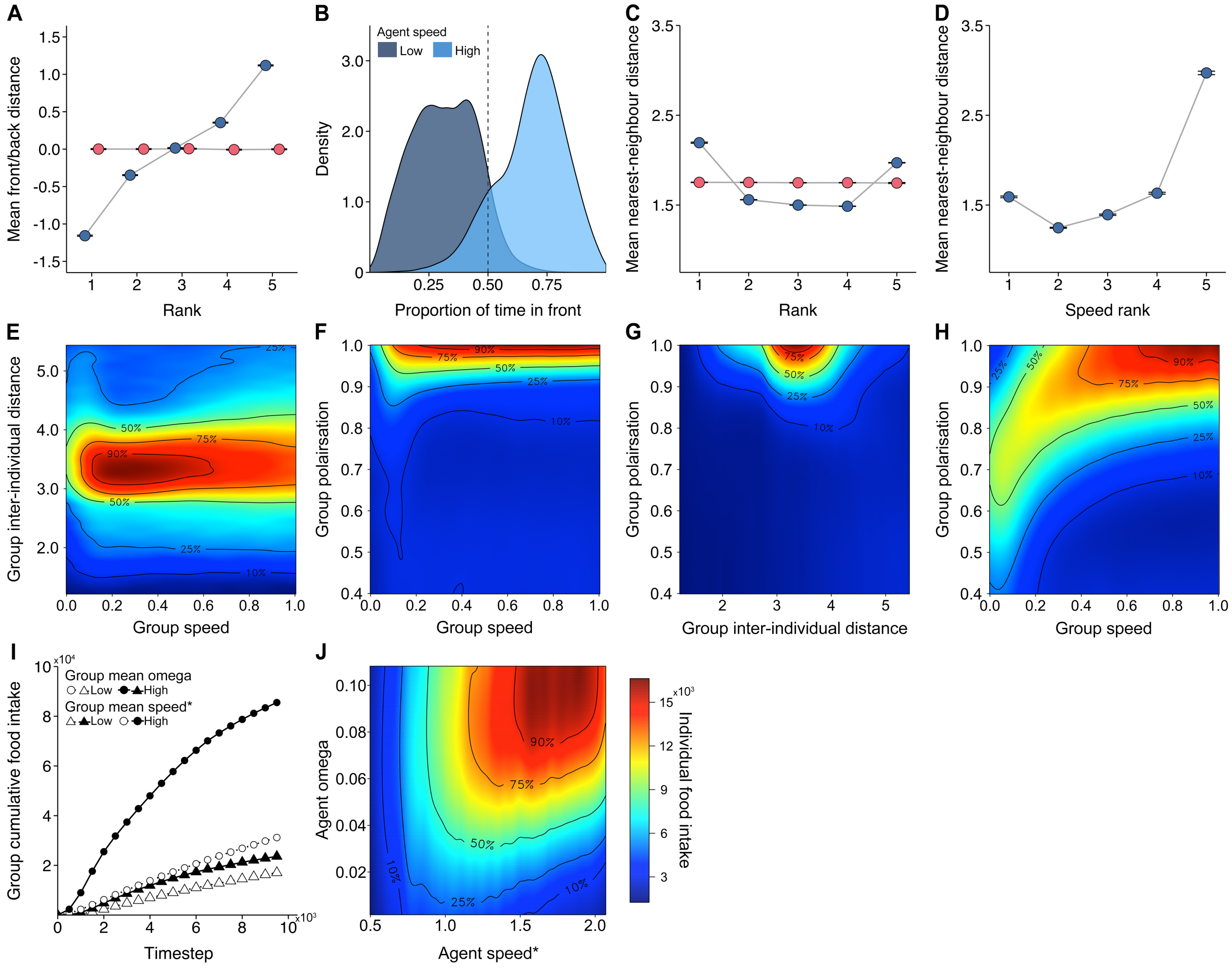

Figure S4. Data from the individual-based model simulations. Related to Figures 2, 3 and 4. (**A**) Mean distance in front/behind the group centroid in terms of an individual's speed rank (blue) or omega rank (goal-directedness; red) in the group (lower numbers indicate higher speed/omega). (**B**) Density plot of the proportion of time individuals spent in front of the group centroid in terms of their set speed, categorized in three equally sized bins with the intermediate bin not shown for clarity. (**C**) Mean nearest-neighbour distance in terms of an individual's speed rank (blue) and omega rank (red). (**D**) Mean nearest-neighbour distance in terms of an individual's speed rank but now with speed scores drawn from a Gamma distribution, see Methods and Figure S1. Front/back and nearest-neighbour distances were averaged across the simulation and expressed in units of repulsion radius. Error bars indicate 95% confidence intervals of the mean. These plots indicate that within a group, faster individuals tend to be towards the front of the group and further from their neighbours (note, also very slow individuals tend to be further away), especially when the distribution of individual speeds is right-tailed. Individual goal-directedness (omega) had no effect on these behaviours. Compare with Figure 2 and see Figure S1. (**E-H**) Surface plots depicting the relationship between (**E**) group speed and cohesion, (**F**) group speed and polarisation, (**G**) group cohesion and polarisation for groups of five individuals, and (**H**) group cohesion and polarisation for groups of 20 individuals. Plots are based on the full dataset but cropped to show the most relevant area only (respectively 90.3%, 90.4%, 92.2% and 85.8% of the full parameter space). Colour scale is square-root transformed and reflects z-scores in percentage relative to the highest bin, with contours representing iso-levels. Plots (**A-H**) are based on 400 replicates of 2,000 time steps taken at intervals of 200 time steps. Plots (**E-H**) indicate that faster groups, i.e. those composed of individuals with higher set speed, were sparser and more polarised than their slower counterparts. The link between speed and polarisation becomes especially clear when the group is larger, with groups of 20 needing higher speed to reach the same level of polarisation. The effect of speed on inter-individual distance, however, is weak. This is partly due to the three zone model, which allows for stable existence of neighbours in the alignment/orientation zone alone (see Methods), and potentially not having variable speed. Compare with Figure 3 and Figure S2. (**I**) Cumulative food intake over time, showing mean values for groups evenly split into four categories based on the average of the set distribution of speed and goal-directedness (omega). (**J**) Surface plot showing individual food intake calculated as the number of food particles consumed by an individual in terms of its speed and goal-directedness (omega). Data was cropped to show the most relevant area (73.6% of the full dataset). For comparison with fish' social proximity tendency (in Figure 4E), symbols of group speed (**I**) are inverted. Plots (**I,J**) are based on 400 replicates of 10,000 time steps taken at intervals of 500 time steps and indicate an interaction between individual's movemens speed and goal-orientedness drove both group and individual foraging performance: groups depleted the food more quickly the faster and more goal-oriented they were, and within groups, individuals that were faster and more goal oriented consumed more food. This is linked to the fact that faster individuals are more in front and therefore arrived at reward sites sooner than their group mates, while omega determined an individual's directedness towards the food once within the cue detection radius (see Methods). Compare with Figure 4.
